# A Decentralized Compositional Framework for Dependable Decision Process in Self-Managed Cyber Physical Systems

**DOI:** 10.3390/s17112580

**Published:** 2017-11-09

**Authors:** Peng Zhou, Decheng Zuo, Kun-Mean Hou, Zhan Zhang

**Affiliations:** 1 School of Computer Science and Technology, Harbin Institute of Technology, Harbin 150001, China; zhoupeng@ftcl.hit.edu.cn (P.Z.); zuodc@hit.edu.cn (D.C.); 2 LIMOS, UMR 6158 CNRS, Université Clermont Auvergne, BP 10125, 63173 Aubière CEDEX, France; kun-mean.hou@uca.fr

**Keywords:** cyber physical system, dependability, flexibility, self-management, scalability, relative time model, composability and compositionality, decentralized decision process

## Abstract

Cyber Physical Systems (CPSs) need to interact with the changeable environment under various interferences. To provide continuous and high quality services, a self-managed CPS should automatically reconstruct itself to adapt to these changes and recover from failures. Such dynamic adaptation behavior introduces systemic challenges for CPS design, advice evaluation and decision process arrangement. In this paper, a formal compositional framework is proposed to systematically improve the dependability of the decision process. To guarantee the consistent observation of event orders for causal reasoning, this work first proposes a relative time-based method to improve the composability and compositionality of the timing property of events. Based on the relative time solution, a formal reference framework is introduced for self-managed CPSs, which includes a compositional FSM-based actor model (subsystems of CPS), actor-based advice and runtime decomposable decisions. To simplify self-management, a self-similar recursive actor interface is proposed for decision (actor) composition. We provide constraints and seven patterns for the composition of reliability and process time requirements. Further, two decentralized decision process strategies are proposed based on our framework, and we compare the reliability with the static strategy and the centralized processing strategy. The simulation results show that the one-order feedback strategy has high reliability, scalability and stability against the complexity of decision and random failure. This paper also shows a way to simplify the evaluation for dynamic system by improving the composability and compositionality of the subsystem.

## 1. Introduction

Once the concept of the Cyber Physical System (CPS) was first proposed by the American National Science Foundation (NFS) in 2006, it soon became so popular that CPS is even regarded as a next revolution of technology which can rival the contribution of the Internet [1]. CPS applications are being explored in various areas, e.g., smart transportation, smart cities, precision agriculture and entertainment. A CPS is a (large) geographically distributed, close-loop system. It closely interacts with the physical world by sensing and actuating. Roughly speaking, a CPS consists of wireless/wired sensor networks (WSNs), decision support systems (DSSs), networked control systems (NCSs) and physical systems/elements. To integrate these four kinds of subsystems, the framework/model of CPS should support both the discrete models (i.e., WSN, DSS and some NCS) and continuous models (i.e., some NCS and physical systems), and integrate them seamlessly.

In the last decades, numerous conceptual frameworks/models were proposed to explore the CPS design in different domains and improve the X-abilities of CPS. One famous proposal among them is the National Institute of Standards and Technology (NIST) reference framework, which was proposed by the CPS Public Working Group of NIST in 2014. This proposal comprehensively analyzes the requirements from various aspects and highlights research issues of *complexity*, *timing*, *adaptability*, *safety*, *reliability*, *maintainability* [2]. However, these conceptual frameworks/models mainly focused on the requirements and the possible models for CPS design, few of them discussed the dependability of CPS [3,4]. Meanwhile, most of the current models or solutions are limited to static centralized architectures, which are incapable of analyzing the dynamic behavior in uncertain environments [3], let alone achieve synergy between adaptability and dependability under the complex constraints.

Complexity is one key challenge to dependability engineering. To design a dependable system, one key is using simplicity to control complexity [5]. Considering the infinite scenarios of a changeable environment, it is infeasible to do a complete testing/simulation to evaluate the decision in design period. Model@run.time is a smart solution to evaluate decision at runtime. Compared to testing/simulation in design period, Model@run.time can significantly narrow the possible testing space, which can decrease the complexity and improve the accuracy of evaluation [6]. In some sense, modeling and evaluating at runtime is also a kind of solution for decision making. Moreover, these decisions should be transformed into executable applications. However, runtime transforming introduces extra complexity, which decreases the dependability. Thus *a systemic solution is needed to build an executable decision to avoid the runtime transforming.*

Rammig proposed the challenges of autonomic distributed real-time systems from: (1) modeling dynamically reconfigurable systems; (2) dynamically reconfigurable system; (3) dynamically reconfigurable target hardware [7]. CPS suffers the same challenges, but more serious. As a CPS consists of various heterogeneous subsystems, the processing behaviors of one same decision are different in most cases, e.g., the execution duration of programs, the reliability of subsystems and the accuracy of data. Moreover, the available solutions for reconstruction are infinite. It is too heavy for Model@run.time solutions to evaluate the inconsistent behavior of these solutions in time. As Rammig argued, the only chance to cope with the challenges of autonomic distributed real-time systems is to enforce composability and compositionality (C&C) [7]. Improving the C&C of subsystems is a promising solution for dynamic reconstruction and runtime evaluation [8,9]. High C&C subsystem design can also comprehensively reduce the complexity of CPSs, simplify the evaluation of decisions and improve the dependability of decision processing.

### 1.1. Overview of the Pattern of Self-Managed Loop

Autonomic computing (AC) is a common trend solution for complex systems [10]. AC systems try to simplify management and improve the adaptability by applying *MAPE-K* methods [10]. In other words, an AC system tries to automatically make decisions (a.k.a. planning) and take actions (a.k.a. executing) at runtime. Self-management is a more detailed proposal for AC systems, which includes four subcategories: *self-configuring*, *self-healing*, *self-optimizing* and *self-protecting* [11]. More and more studies try to introduce self-management (or AC) into CPS to improve the flexibility and adaptability [12,13], and the dependability [14,15]. Warriach et al. expanded the categories of self-management with a set of self-* abilities, including *self-healing*, *self-protection*, *self-awareness*, *self-organizing*, *self-synchronization* and *self-configuration*, and analyzed both functional and nonfunctional requirements of self-healing in smart environment applications [16].

**Figure 1:**
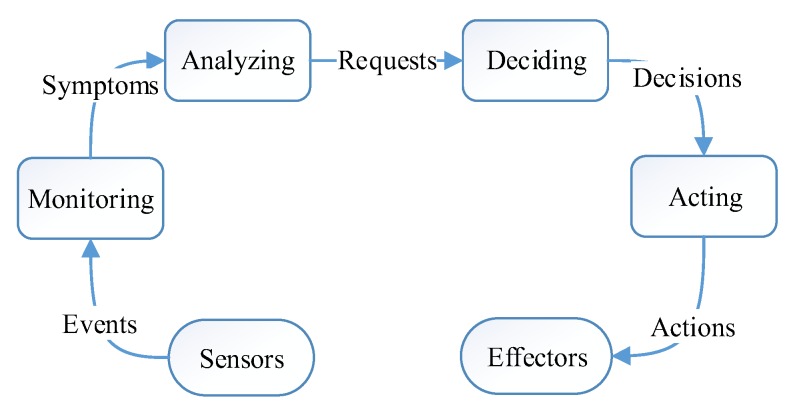
The system-centric self-management process of AC Systems.

Generally, all these self-* processes of the AC system involve four main phases: monitoring, analyzing, deciding, and acting [17], which is illustrated in Figure 1. AC was once proposed for business computer systems, which are generally deployed in a well-protected environment (such as data centers), and rarely affected by the natural world. Hence, a general AC system doesn’t need to adapt to the changeable external environment. *The self-management for the general AC system is system-centric*, which mainly focuses on the continuous improvement of system itself, such as load balance, reducing resource consumption of services, improving dependability and security of system. Indeed, it is us, human beings, who are trained to adapt to the AC systems.

**Figure 2:**
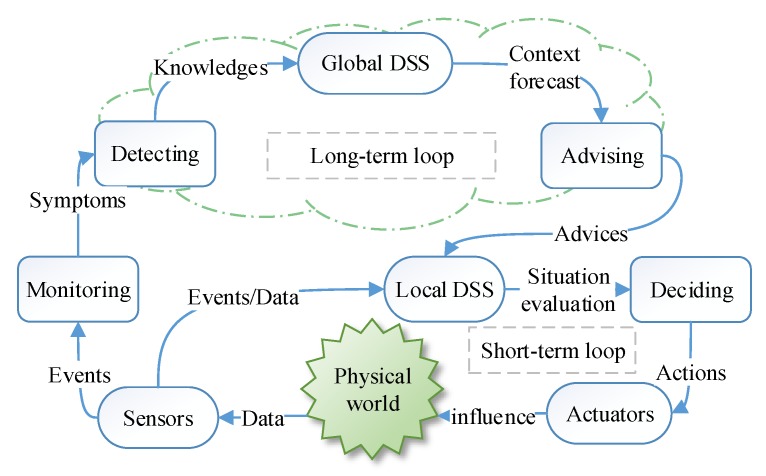
The environment-in-loop self-adaptation process of CPS.

Compared to general AC system, a self-managed CPS has to interact automatically with the physical world. To behave properly, it should take the right actions in the right place, at the right time, with reasonable processing speed. In other words, it should not only continuously improve the system itself, but also adapt to the variable environment. Hence, a self-managed CPS should form two types of closed loops [13,18,19]. One is a self-healing loop [19], which is similar to the schema in Figure 1. Another is the interactive loop between the cyber world and the physical world, which is illustrated in Figure 2. The interaction loop includes long-term loops for causal reasoning (big data driven *MAPE-K loop*) and short-term loop for dependable decision process (the feedback control loop). The self-healing loop and interaction loop may influence each other, e.g., the temperature rise will trigger the cooling control loop (environment-in-loop adaptation) and also affects the reliability of hardware (system-centric self-management). In this paper, we focus on improving the consistency of event observation (the long-term loop) and improving the dependability of decision process (the short-term loop).

Centralized decision arrangement is the most common solution for AC systems. The processing flow of a decision is controlled by a (local) central system, such as DSS. *The processing flow fails if the decision manager fails* (a.k.a. single point of failure). The dependability of such processing solutions is limited by the central system. To overcome this issue, one generic solution is deploying redundant decision arrangement system. However, it may generate conflicting decisions because two redundant decision control systems may have the inconsistent observation results and get different events’ orders.

Even to one same centralized decision manager, the order of events may be wrong. The physical events occur in parallel and sensors are distributed in CPS. Due to various issues (e.g., errors, failures, delays, etc.) [20,21], the clocks of sensors may not be precisely synchronized. As a consequence, different sensors may generate inconsistent timestamps for one same event, which confuses the DSS and misguides the fault diagnosis methods. Taking precision agriculture as an example, the DSS analyzes the soil moisture with the current temperature and the status of leaves; and then makes a final decision that the plants could and should be watered. Then the nozzle starts to spray water at the timestamp $t_{1}$, and the event of starting to spray is denoted as $e_{1}$. The soil moisture sensor detects the increase of humidity at the timestamp $t_{2}$, and this event is denoted as $e_{2}$. If we hold the assumption of the global reference time, it implies that $t_{1}$ and $t_{2}$ are comparable. When DSS receives $e_{2}$ before $e_{1}$ and finds that $t_{1}>t_{2}$, the DSS will alarm that there are some things wrong with the nozzle or pipe (i.e., leaking). Whereas in a real multi-agent CPS, the timestamps $t_{1}$ and $t_{2}$ are not comparable because of the time synchronization deviation between the node with soil moisture sensor and the actuator with nozzle. Hence, the timing order of $e_{1}$ and $e_{2}$ is indistinguishable. Consequently, the information of causality between physical events is lost, and further analysis becomes impossible. The timing issue is challenging the correctness of self-managing decisions, especially to the real-time CPS.

Another challenge is guaranteeing the consistency of the dynamic behavior with simple and dependable (Model@run.time) solutions. As a kind of system of systems (SoS), a CPS is composed of numerous heterogeneous subsystems. These subsystems may also recursively consist of various other subsystems. To describe this feature, we should abstract the subsystems with a model that is closed under composition. To model the dynamic structure and dynamic behavior for self-management, the reference framework should be flexible enough to describe the runtime composition. To guarantee the quality of decisions and quantitative analyze the dynamic behavior, the properties of subsystems should be composable and the requirements of decision should be runtime decomposable. With systematic consideration of these requirements, we proposed a framework based on compositional actors.

The contributions of the paper are manifold. We introduce a relative time solution to solve the inconsistent event observation in CPS, which forms a foundation for a decentralized decision process. Moreover, we design a formal compositional framework of the decentralized decision process. A self-similar recursive actor interface is proposed to simplify self-management. We analyze the composability and compositionality of our design and provide seven composition patterns. A one-order dynamic feedback strategy is introduced to improve the reliability, scalability and stability of decision process.

### 1.2. Structure of Paper

**Figure 3:**
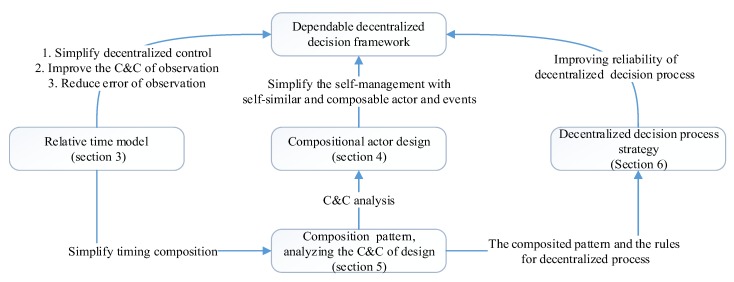
The relationship of the work.

The remainder of the paper is organized as follows: Section 2 is about the related works on self-management CPS and formalization. Section 3 introduces the relative time model to guarantee the consistency of event observation and the qualitative contrastive analysis with the absolute time model. Section 4 details the actor-based formal model and the interface design. We analyze the composability and compositionality of reference framework in Section 5. We introduce a simple decentralized decision process strategy and one-order feedback decentralized dynamic decision process strategy and compare the reliability with other two strategies in Section 6. The relationship of Section 3, Section 4, Section 5 and Section 6 is shown in Figure 3. Section 7 is a case study of the dependability of decentralized decision process. Section 8 draws the conclusions.

*Notations*: (1) without additional notes, we use t or $t_{b}$ to represent the absolute timestamp, τ to represent the duration, $t_{l}$ and $t_{l}+\tau$ to represent the relative timestamp in the remainder of our paper. We use $\hat{\tau }$ to represent the static duration for WCET, BCET, and static requirement of advice, and τ to represent the real duration or the dynamic requirement of decision; (2) the term “subsystem” is an agent with several actors. “Subsystem” to decentralized CPS is the “component” to centralized system; (3) the “decision” in this paper is a dynamical concept, which is similar to the concept of “application” and “program”. An example is introduced in Appendix A.1 for further understanding of the decentralized decision process.

## 2. Related Works on Self-Management Framework for CPS

Roughly speaking, a CPS has to face two kinds of uncertainty. One is the changeable environment, another is the unpredictable process flow caused by resource competition and random failures. To behave properly under uncertainty, CPS should make and process decisions according to the context. For each self-adapting decision, CPS should select the right subsystems from various heterogeneous candidates, organize them in the right way, and coordinate the decision process on subsystems. For self-healing, each prearranged subsystem may be replaced by others at runtime, and heterogeneous redundant subsystems should cooperate together to improve the reliability. No matter self-adapting or self-healing, CPS has to dynamically reconstruct its services, structure and topology at runtime. However, dynamic reconstruction decreases the controllability and predictability of CPS behavior. It is a big challenge to achieving the consistent quality of decisions process, such as consistent timing, predictable reliability and safety. To overcome these issues, systemic solutions are need to evaluate the correctness of reconstruction and to guarantee the consistency of the dynamic behavior of decisions.

A good framework is the foundation for self-management CPS. Massive aspect oriented formal framework have been published to improve the functional performance of CPS [22,23], and various frameworks are proposed for self-adapting CPS. As we classified in the survey [3], these frameworks of CPS can be classified into three types: Service Oriented Architecture (SOA)-based frameworks, Multi-Agent System (MAS)-based frameworks, and other aspect oriented frameworks. Compared to SOA-based frameworks, MAS-based frameworks are more lightweight and more scalable. As a kind of SoS, CPS shows high flexibility, but low predictability. More and more researchers are paying attention to verification and validation (V&V) of the dynamic structure and behavior of CPS with Model@run.time methods to improve their predictability. A formal framework is an alternative solution to improve the predictability and dependability without introducing too much complexity.

Unfortunately, there are relatively few studies on formal framework (architecture) and dependability evaluation [3]. SCA-ASM is a formal SOA-based framework for modeling and validating distributed self-adaptive applications. SCA-ASM can model the behavior of monitoring and reacting to environmental changes and to internal changes, and the related operators for expressing and coordinating self-adaptive behaviors [24,25]. A MAS-based framework based on the logic-based modeling language called SALMA was introduced, this model focuses on the information transfer processing [26]. In the domain of cyber-physical transportation, Mashkoor et al. built a formal model with higher-order logic [27]. These frameworks are based on centralized decision process control solutions. The centralized decision controller is slow in reacting because of the long transmission delay, which increases the safety risk. Moreover, the centralized decision controller is a single point of failure. Decentralized control can overcome these drawbacks, and more and more studies are being published in this field. A formal framework was proposed for decentralized partially observable Markov decision process problems. Based this framework, a policy iteration algorithm is presented to improve the coordination of distributed subsystems [28]. A decentralized control solution based on Markov decision processes is proposed for automatically constructed macro-actions in multi-robot applications [29]. However, these solutions mainly focus on the performance and convergence speed, the dependability and timing issues are rarely discussed. Moreover, these researches are based on ideal subsystems assumption, where all subsystems are dependable and behave consistently.

In the real world CPS, numerous heterogeneous subsystems are applied. These subsystems have different properties, i.e., different performance, different precision, which complicate the control of decision process. Various kinds of solutions have been invented to hide the differences between subsystems, such as middleware and virtualization [30,31], interface technology [32] and specification [33]. These technologies simplify the self-adaptation by providing consistent interfaces. Nevertheless, it is still not enough for self-management CPS. For safety, the risk of all decisions should be evaluable, and all actions should be predictable, which implies that all services and actions have consistent, stable behavior at run-time. Specifically, a CPS should be stable in the timing behavior and the reliability of services, and the accuracy of data, etc. Otherwise, the inconsistent and uncontrollable behaviors will make the CPS unpredictable and increase the risk of safety, mislead the DSS into making wrong decisions, i.e., the reasoning failure caused by inconsistent timestamp, which was introduced earlier.

To hide the differences and guarantee the quality of services, one promising way is to improve the C&C of services. Composability is the property whereby component properties do not change by virtue of interactions with other components [9]. It comes from the philosophy of reductionism, which highlights the consistent behavior of the component when it cooperates with other components to build a whole system. On the contrary, compositionality is originated from holism. Compositionality is that system level properties can be computed from and decomposed into component properties [9]. It is more about the capacity of decomposition of the system level properties. It focuses on the consistency between the system level properties and its divided properties (component properties), where the system level properties can be calculated with components/subsystems properties. For more detailed discussions about composability and compositionality readers may refer to [9]. By the way, the concepts of composability and compositionality are interchangeable in some studies.

Designing subsystems with high C&C can reduce the complexity of CPS and systematically improve the quality of services. A theory of composition for heterogeneous systems was proposed to improve the stability, which decouples stability from timing uncertainties caused by networking and computation [34]. Nuzzo introduced a platform-based design methodology based on contracts to refine the design flow. This methodology uses contracts to specify and abstract the components, then validates contracts according to the structure of CPS in design period [35]. An I/O automata-based compositional specification theory is proposed to abstract and refine the temporal ordering of behavior, and to improve the reasoning of the behavior of components [36], which is useful for dynamic decision evaluation and fault diagnosis. To guarantee the timeliness of real-time operations, a formal definition of timing compositionality is introduced [37]. However, how to guarantee in general the quality of dynamic characteristics such as timing [37], safety [34] and dependability is still an open issue. Both new architectures and evaluation methods are needed for guaranteeing the timing and the dependability at runtime. A contract-based requirement composition and decomposition strategy was introduced for component-based development of distributed systems [38]. This work is a valuable reference to the solution design for Model@run.time-based decision evaluations.

To achieve dependable CPS, systematic solutions are necessary. Both traditional means and self-healing methods are useful for maintaining the dependability of CPS. These methods should be applied organically at different levels to achieve dependability without introducing too much complexity. A satellite oriented formal correctness, safety, dependability, and performance analysis method is introduced in [39]; it comprehensively applies the traditional methods to improve a static architecture, yet the traditional means are limited to static architectures, and they become less and less efficient for CPS [3]. Self-healing methods are the trend to manage the dependability of the dynamic structure, which generally adjusts the architecture to prevent or recover from failures with flexible strategies. A simplex reference model was proposed to limit the fault propagation in CPS that built with unreliable components [40]. A methodology is introduced to formalize the requirements, the specification and the descriptive statements with domain knowledge, it shows a systematic solution to verify the dependability of CPS with formal models [41].

What’s done cannot be undone, so this hardly eliminates the negative effects of a wrong physical operation, which makes great claims upon the dependability of CPS. Without maintenance of services and self-healing solutions, self-adapting CPS is still inapplicable. Considering the complex influence between self-healing actions and self-adapting actions, a good formal framework is needed to simplify the decision evaluation at run-time. To address the complexity, we need a systemic solution to apply self-management without introducing too much complexity.

## 3. Improving the C&C of Timing Behavior with a Relative Time-Based Model

Time is important to computing [42], especially for feedback control and causal reasoning. As a necessary condition for causal reasoning, it is important to achieve consensus on the timing behavior of both physical and cyber events. Moreover, the precise time can improve the control and cooperation between subsystems. Both context aware-based self-adaptation and fault prevention-based self-healing can benefit from the accurate causal reasoning and precise decision control. Hence, it is necessary to eliminate the temporal difference among subsystems and improve the C&C of timing for the self-management CPS.

To improve the C&C of timing and make all events observers achieve consensus on timing behavior (the same order of observed events). One intuitive solution is to establish a global reference time with a precisely timed infrastructure and time synchronization protocol. A time-centric model has been introduced for CPS [43]. It is a global reference time-based solution where every subsystem shares one absolute reference time. It is relatively easy to meet the assumption of global reference time for wired small scale CPS. Whereas for a large scale wireless connected CPS, such as the smart transportation CPS and the precision agriculture CPS, maintaining the consistent reference time (absolute time) is a big challenge [20,21].

Furthermore, even if we have a well synchronized system, it still can’t achieve consistent absolute time and reproduce the causal relationship of events in cyberspace due to the imprecise timestamp. In fact, the timestamp of an observed event is rough. The accuracy of a timestamp depends on the sensitivity of the sensor, the processing speed, the sampling period, even the distance between the target object and sensor. Imagine that a physical event occurs at timestamp $t_{p}$ and the sensor detects the event at timestamp $t_{s}$, where $t_{p}$ and $t_{s}$ are absolute times, $t_{p}<t_{s}$ (because sensing takes time). To sensors (especially to the smart sensors integrated complex data analysis), $t_{s}-t_{p}$ is not equal on different subsystems because of the sensitivity and the processing speed. Even to one same sensor, $t_{s}-t_{p}$ is under stochastic volatility. Consequently, it’s impossible to get the consistent absolute time of events in distributed CPS.

As current causal analysis methods just need the order of events, absolute time is an overly restrictive conditions, e.g., for logical reasoning $e_{1}\wedge e_{2}\to r$: if two events $e_{1}$ and $e_{2}$ occur then we must have the result (event) r; or for quantum causal analysis with probability $P(r|e_{1})$: the probability of the observing event r given that event $e_{1}$ is true/observed. Few technologies support to deduce further conclusion from the accurate time difference $\Delta t_{r\to e}$ between the result event r and the event e. There are two main reasons: (1) as $\Delta t_{r\to e}$ is affected by many factors, the acceptable range of $\Delta t_{r\to e}$ maybe too large. It is difficult to quantitatively analyze the stochastic volatility of $\Delta t_{r\to e}$. For example, it takes several weeks to observe the effect of fertilization. In the meantime, various factors may changes the efficient of fertilizer; (2) meanwhile, most events are irrelevant, it wastes resources to guarantee the absolute time of these events.

In general, there are two kinds of timekeeping methods. One is absolute time, where all subsystems share the same reference time (i.e., UTC) and the timestamp of event $t_{b}$. Another is based on local time, where all subsystems have a different local reference time $t_{l}$. For one same event, these subsystems have different observation timestamps $t_{l}+\tau$. Analyzing the sequence of events is the first step for mining the relationship between events. The common method to get the order is calculating the timestamp difference Δt between two events. With absolute time, we can directly get the difference $\Delta t_{ab}=t_{b2}-t_{b1}$. With local time, it is relatively complex. As the base reference time $t_{l}$ is different, the common solutions of local timestamps are not directly comparable. The difference of two reference times $\Delta (t_{l2}-t_{l1})$ is necessary, so the final timestamp difference of two events is $\Delta t_{rf}=\tau_{2}-\tau_{1}+\Delta (t_{l2}-t_{l1})$.

**Figure 4:**
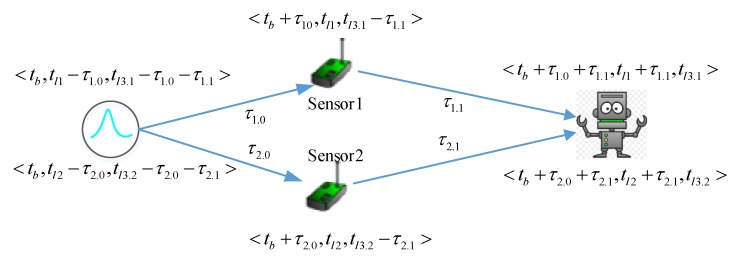
The observation timestamp of an event in different observers’ view.

As observation is relative to each observer and each case, we propose a relative time model. Every subsystem just needs to record the duration that it takes to observe the event. The relationship of absolute time and different observers’ timestamp is depicted in Figure 4. The tuple of timestamp is (absolute time, timestamp according to sensors’ view, timestamp according to actuator’s view). For example, a physical event occurs at the absolute time $t_{b}$. It takes *sensor1* $\tau_{1.0}$ to observe the physical event, and the absolute timestamp is $t_{b}+\tau_{1.0}$. The actuator observes the event from *sensor1* at $t_{b}+\tau_{1.0}+\tau_{1.1}$. Here, let us assume that *sensor1, sensor2* and actuator are not well synchronized, they have to record the observation based on their own local times. The timestamp in local time when *sensor1* observes the event is $t_{l1}$, where $t_{l1}$ and $t_{b}+\tau_{1.0}$ are two timestamps based on different reference times, and $t_{l1}=t_{b}+\tau_{1.0}$. Obviously, *sensor1* can infer that the physical event occurs at $t_{l1}-\tau_{1.0}$. Likewise, the actuator observes the event from *sensor1* at absolute timestamp $t_{b}+\tau_{1.0}+\tau_{1.1}$, and at $t_{l1}+\tau_{1.1}$ from sensor1s’ view, and at $t_{l3.1}$ from the actuator’s view. The event from *sensor2* occurs at $t_{b}+\tau_{2.0}+\tau_{2.1},\;t_{l2}+\tau_{2.1},\;t_{l3.2}$, where $t_{l3.1}$ maybe not equal to $t_{l3.2}$. As mentioned earlier, we can’t figure out the order of two observations based on the timestamps $t_{l1}$ and $t_{l2}$. To simplify the calculation of $\Delta t_{rf}$, one intuitive solution is to select a good observer to let $\Delta (t_{l2}-t_{l1})=0$. The actuator is such an observer, the actuator can infer that the event occurs at $t_{l3.1}-\tau_{1.0}-\tau_{1.1}$ and $t_{l3.2}-\tau_{2.0}-\tau_{2.1}$. As $t_{l3.1}$ and $t_{l3.2}$ share the same local reference time, we have the difference of the timestamps $\Delta t_{rf}=t_{l3.1}-\tau_{1.0}-\tau_{1.1}-(t_{l3.2}-\tau_{2.0}-\tau_{2.1})=(t_{l3.1}-t_{l3.2})-((\tau_{1.0}+\tau_{1.1})-(\tau_{2.0}+\tau_{2.1}))$, where $\tau_{1.0}+\tau_{1.1}$ and $\tau_{2.0}+\tau_{2.1}$ are amount of the process time and transmission time. Currently, *the best observers are the sensors. To achieve this, we propose a dynamic decision process framework, which will be introduced in the last part of this paper*.

**Figure 5:**
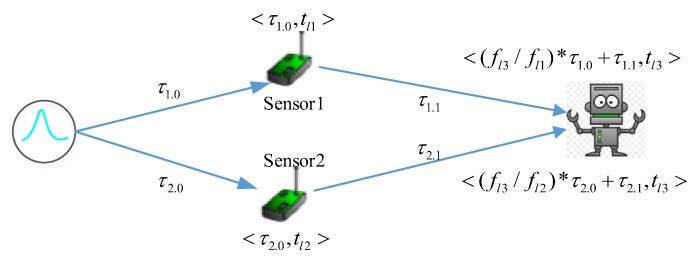
Relative time model with frequency revised.

Theoretically speaking, two observations of one same event should be identical. For timing, two observations should have the same timestamp, where $\Delta t_{rf}=0$. However, it may be not true in the real world system, because the clocks on different subsystems have different speeds due to the frequency deviation of oscillators. The revised relative time model is shown in Figure 5, where f is the system clock frequency of the respective subsystems. The tuple of timestamp is (accumulated time from the physical event is generated, local time). For example, the actuator observes the event from *sesnor1* at local time $t_{l3}$, the accumulated time is $(f_{l3}/f_{l1})\times \tau_{1.0}+\tau_{1.1}$. From the view of the actuator, the physical event is generated at the relative time $t_{l3}-(f_{l3}/f_{l1})\times \tau_{1.0}-\tau_{1.1}$.

**Figure 6:**
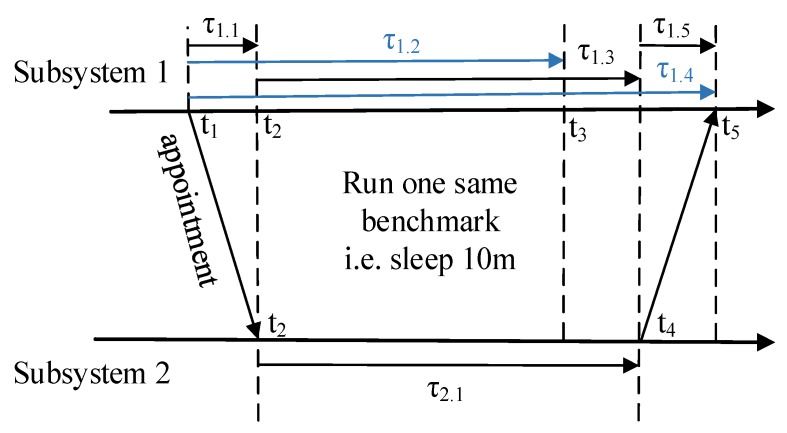
Appointment and execution method for relative frequency scale calculation.

So far, the remaining problem is how to automatically get the scale of the frequency $f_{l2}/f_{l1}$. Based on the time synchronization solutions for the symmetric network [21] or the asymmetry network [44], every observer can get the duration of transmission time by exchanging message methods according to its own clock, whereas compared to the absolute time model, the relative time model doesn’t need to synchronize the clocks. Instead, the neighbor subsystem just needs to check the scale of the frequency $f_{l2}/f_{l1}$. We design an appointment and execution method to calculate $f_{l2}/f_{l1}$ which is shown in Figure 6. For easy understanding, all the timestamps in Figure 6 are absolute times, and all the duration are relative. With the exchanging message method, every subsystem has already got the $\tau_{1.1}$ and $\tau_{1.5}$ (actually, $\tau_{1.1}$ and $\tau_{1.5}$ can be inaccurate because of $\tau_{1.1}+\tau_{1.5}\ll \tau_{1.2},\;\tau_{1.1}+\tau_{1.5}\ll \tau_{1.3}$).

At the beginning, *subsystem1* makes an appointment with *subsystem2* to execute one same benchmark at the same time (the execution speed of the benchmark should be independent to the hardware architecture, i.e., the size of cache). *Subsystem1* takes $\tau_{1.2}$ to finish the benchmark and takes $\tau_{1.4}$ to get the finished signal from *subsystem2*. From the view of *subsystem1*, it takes *subsystem2* $\tau_{1.3}=\tau_{1.4}-\tau_{1.1}-\tau_{1.5}$ to finish the benchmark. Thus $f_{l2}/f_{l1}=\tau_{1.2}/(\tau_{1.4}-\tau_{1.1}-\tau_{1.5})$. We can simplify the relative time model by calibrating the clock (oscillator) of all subsystems with a base clock (oscillator) before deployment, and set $f_{b}/f_{l}$ for every subsystem and get an *absolute duration*. To simplify the formulation, we use the term *absolute duration* in the remainder of this paper. We can easily change the *absolute duration* $\tau_{b}$ to relative duration $\tau_{r}$ with formulation $\tau_{r}=(f_{l}/f_{b})\times \tau_{b}$ if it is necessary.

Considering related physical events are in geographical proximity, the observer should be as close as possible to the source of events. Thus, the accumulated error of duration of two events will not be too large that CPS can’t reproduce cause-effect relationships. As soon as the events being observed and serialized, their orders (relationships) are confirmed. Then, CPS can apply various technologies for further analysis of the relationship between these events.

The relative time model records the duration of events instead of the absolute timestamp when events occur. Ideally, $f_{l2}/f_{l1}$ just needs to be set once. In the real world system, it may still need to be calibrated several times during the system lifetime, because oscillators are affected by temperature and aging. Anyhow, this process can decrease the frequency of synchronization significantly and has a more stable error. Furthermore, the relative time model doesn’t need a global reference time, which can improve the scalability of CPS significantly, no matter the subsystems are heterogeneous or not. Detail comparison between relative model and absolute time mode is beyond the scope of this paper, the qualitative conclusion is shown in Table 1.

## 4. The Formal Reference Framework for Decision Process

As mentioned earlier, it can benefit a lot by improving the C&C of subsystems. Our solution mainly focuses on *the composition of subsystems* and *decomposition of requirements at run-time*. In this section, we introduce the formal reference framework for the dynamic decision process.

### 4.1. Overview of the Actor Based Framework

A self-managed CPS should automatically sense the environment and diagnose itself, then make both self-adapting and self-healing decisions, and execute these decisions. Decision making and executing are the two key parts to form the close-loop. Improving the C&C of subsystems can decrease the complexity of the process of both decision making and decision executing. Composability can simplify decision making by simplifying the evaluation of the reasonability of decisions. Otherwise, DSS has to enumerate all available combinations. Compositionality can simplify decision executing by simplifying the decomposition of the requirements at runtime, which is helpful for guaranteeing the dependability of decision execution. Composability and compositionality are two sides of the same coin, which are the necessary qualities for a good CPS framework.

**Figure 7:**
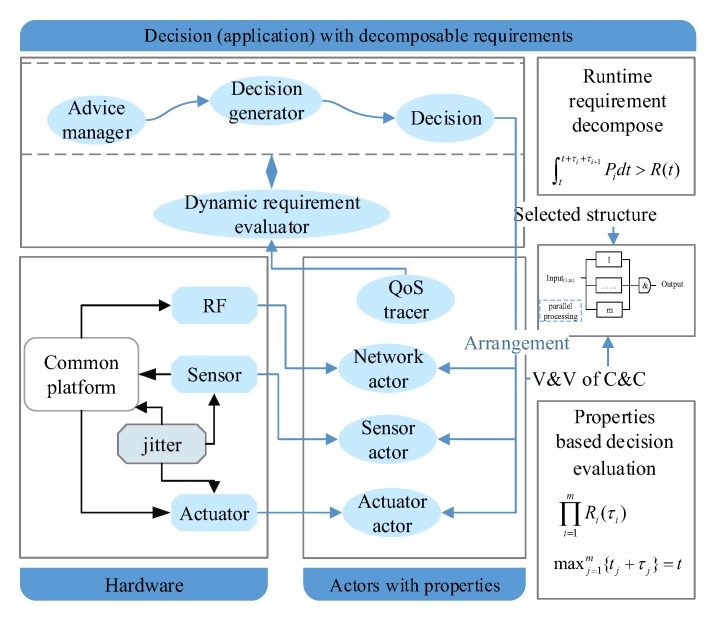
Actor-based decision evaluation and self-management based on requirements management (RF: Radio Frequency devices).

A self-management CPS includes two parts: (1) the agent platform, which includes hardware and corresponding actors; (2) the dynamic behavior management subsystem (decision subsystem). An overview of an actor-based framework for a self-management CPS is shown in Figure 7. The actor is the atomic abstraction of subsystems in our reference model. Agents are the platform for decision execution. The behavior of a decision depends on both the properties of hardware and the properties of the actors. To simplify, we integrate the hardware properties into the properties of the actors. We assume that the decision has been made by the DSS. Here, we focus on the evaluation of advice and the dependability guaranteeing in run-time.

### 4.2. Actor and Decision Formalization

**Definition 1** (Actors)**.** *An actor is a time bounded Mealy finite state machine (FSM)* $Actor=(Tid,\Sigma ,S,s_{0},\Theta ,\Psi ,T)$*. Where* Σ *is a finite set of events, and event should not be empty* ε∉∑*,* $e_{timer}\in \sum$ *is the timer interrupt event based on local time;* S *is a finite set of states, where* ∀s∈S *has a time bound* $\tau_{s}$ *that represents the duration that the actor stays in state* s*, and the maximal time bound of state* s *denotes* $s|{\hat{\tau }}_{s}$*;* $s_{0}\in S$ *is the initial state, and* $s_{0}|{\hat{\tau }}_{s_{0}}=+\infty$. Θ⊆S×Σ×S *is a set of transition operations, where* $\theta_{k}=s_{k}\times e\to s_{m}|{\hat{\tau }}_{\theta }^{k},\theta_{k}\neq \phi ,\Theta \neq \Phi$*,* ${\hat{\tau }}_{\theta }^{k}$ *is the time bound of transition* $\theta_{k}$*;* Ψ⊆S×∑×S *is a set of actions, where* $\psi_{k}=s_{k}\times e\to s_{m}|{\hat{\tau }}_{\psi }^{k},\phi \in \Psi ,\Psi \neq \Phi$*. In addition, an actor must contain one non-empty action* ψ*; otherwise, it can’t interact with other actors.* ${\hat{\tau }}_{\psi }^{k}$ *is the time bound of action* $\psi_{k}$*;* T *is the union set of the time bound of the state, transition and action* $T=\{{\hat{\tau }}_{s}\}\cup \{{\hat{\tau }}_{\theta }\}\cup \{{\hat{\tau }}_{\psi }\}$*. Here we have* $\left\{{\hat{\tau }}_{\theta }\right\}$ *and* $\left\{{\hat{\tau }}_{\psi }\right\}$ *because the transition in cyber space and the action with physical world are always asynchronous.* Tid *is the identifier of the type of actor.*

In the rest of this paper, we will simplify the notation by writing $s_{i}\cdots s_{n}$ instead of *trajectory* (the transition sequence) $<\varepsilon_{i,}s_{i},\theta_{i},\psi_{i}>,\cdots ,<\varepsilon_{n,}s_{n},\theta_{n},\psi_{n}>$, whenever the context allows doing so without introducing ambiguity. We define $Actor_{i}=Actor_{j}$ if and only if $Actor_{i}$ and $Actor_{j}$ produce identical output sequence $\psi_{1}\cdots \psi_{n}$ for all valid input sequences $\varepsilon_{i}\cdots \varepsilon_{m}$, where n≤m. $\left(Actor_{i}=Actor_{j})\Leftrightarrow (Actor_{i}.Tid=Actor_{j}.Tid\right)$, $Actor_{i}=Actor_{j}$ just says that the two actors has the same *trajectory*, the two actors may be not isomorphism, and the properties of two actors can be different, i.e., the performance, the reliability etc. If $Actor_{i}=Actor_{j}$, and also all the properties of $Actor_{i}$ and $Actor_{j}$ are the same, we use the notation $Actor_{i}\equiv Actor_{j}$.

Every actor has a set of properties, which we denote as (Actor,P). In our dependable framework, $P=<{\hat{\tau }}^{b},{\hat{\tau }}^{w},p(\tau )>$, where ${\hat{\tau }}^{w}$ is the worst-case execution-time (WCET) of processing a decision, ${\hat{\tau }}^{b}$ is the best-case execution-time (BCET). p(τ) is the failure rate, where τ is the online time or the elapsed time from last recovery. We can calculate ${\hat{\tau }}^{b}$ by replacing the BCET ${\hat{\tau }}_{s}^{}$ and ${\hat{\tau }}_{\theta }^{}$ in the formula $\hat{\tau }=\sum_{s\in S_{p}/\{s_{k}\}}\left({\hat{\tau }}_{s}^{}+{\hat{\tau }}_{\theta }^{}\right)+{\hat{\tau }}_{\psi }^{k}$. Likewise, we have the ${\hat{\tau }}^{w}$.

**Figure 8:**
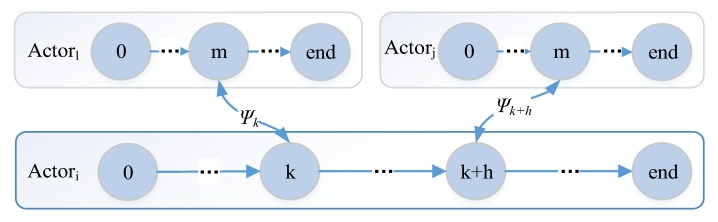
The interaction of actors.

Notice ${\hat{\tau }}^{b}$ and ${\hat{\tau }}^{w}$ are not the time from $s_{0}$ to $s_{end}$. For the case presented in Figure 8, $Actor_{i}$ receives output in state $s_{k}$, and generates a new output in state $s_{k+h}$. Thus $S_{p}=\{s_{k},s_{k+1},\cdots ,s_{k+h}\}$ is the $S_{p}$ in formula $\hat{\tau }=\sum_{s\in \{s_{k+1},\cdots ,s_{k+h}\}}\left({\hat{\tau }}_{s}+{\hat{\tau }}_{\theta }\right)+{\hat{\tau }}_{\psi }^{k}$. We also can get the ${\hat{\tau }}^{b}$ and ${\hat{\tau }}^{w}$ with the Monte Carlo method.

**Definition 2** (Actor Composition)**.** *Let* $CP=(ACT_{p},ACT_{s},M,\to )$ *be a composition, where* $ACT_{s}=\{Actor\}$ *is a set of preorder actors of the composition,* $ACT_{p}\neq \phi$*;* $ACT_{s}=\{Actor\}$ *is a set of successor actors of the composition, and* $ACT_{r}\neq \phi$*;* M *is the messages (special events) set for the composition communication;* $\to =ACT_{p}\times M\times ACT_{s}$ *is the communication pair of the composition, the arrow is the direction of message.*

We use the notations $Actor_{i}(s_{k},\psi_{k})\overset{msg}{\to }Actor_{j}(s_{m},\psi_{m})$ to represent point-to-point communication that $Actor_{i}$ sends a message msg in state $s_{k}$ with action $\psi_{k}$, and $Actor_{j}$ receives msg in state $s_{j}$ with action $\psi_{j}$, and $Actor_{i}\in ACT_{p}$, $Actor_{j}\in ACT_{s}$. $Actor_{i}(s_{k},\psi_{k})\overset{msg}{\to }\{Actor_{j}(s_{m},\psi_{m})\}$ is the one-to-many communication. $\left\{Actor_{i}(s_{k},\psi_{k})\}\overset{msg}{\to }Actor_{j}(s_{m},\psi_{m}\right)$ is the many-to-one communication. The three types of communication are illustrated in Table 2. *Using message-based composition, we can decouple the actors and reduce the constraints of operation interfaces*.

We will use msg(i,j) to represent the communication in short, and $msg_{i,j}$ to identify the message itself. Without explicit mention, msg(i,j) also implies that the $Actor_{i}$ and $Actor_{j}$ have the same definition of the structure of the message; if not, $Actor_{j}$ will ignore the message. In addition, we use $msg_{k,i}=msg_{k,j}$, where $msg_{k,i}$ shares the same description of structure with $msg_{k,j}$, the context/value of message could be different. $msg_{k,i}\equiv msg_{k,j}$ means that $msg_{k,i}$ and $msg_{k,j}$ have the same the structure and the context. $msg_{k,i}≜msg_{k,j}$ means that $msg_{k,i}$ and $msg_{k,j}$ are identical, which means that $msg_{k,i}\equiv msg_{k,j}$ and the properties (e.g., time bound etc.) of message are the same.

Notice that, according to this model, one Actor can send a message to itself, whereas in a real system, only the composited actor can send the message to itself (the subsystem of the composited actor). It is meaningless for an atomic actor to do so. CP is the agent/subsystem level view of interactions. These interactions are not limited to applications’ communication, which include the interactions between agents to maintain the infrastructures, e.g., topology, QoS, etc. For advice evaluation, we ignore the communication for maintenance.

**Definition 3** (Advice)**.** *Let* $Ad=(X_{s},<Tid_{act},X_{f}>,⊗,X)$ *be an advice, where* X *is a set of observation event* $\chi_{o}=<Ob,Tid_{t},\chi_{t}>$*, which represents the preorder actor observes whether the target* $Actor_{tid=tid.t}$ *has generated event* $\chi_{t}$ *or not,* $X=\{\chi_{o}\}\cup \{\chi_{t}\}\cup \sum$*,* $\chi_{o}\cap \Sigma =\Phi$*,* $\sum \subseteq \chi_{t}$*;* Ob *is an composition which includes an operation instruction* op *on event* $\chi_{t}$*,* $Ob=msg(Tid_{s,}Tid_{t}).<op,Tid_{t},\chi_{t}>\in \mathrm{CP}$. $Tid_{act}$ *is the identifier of actuator actor to take the action,* $X_{s}$ *is the action triggering conditions,* $X_{f}$ *is the action finishing conditions,* $X_{s}\subseteq X$*,* $X_{f}\subseteq X$*;* ⊗ *is Boolean operations* {or,and,not}*. A generic form of advice is defined as* $if\underset{\chi \in X_{s}}{⊗}\chi ,\;then\;excute\;Actor_{Tid=Tid_{act}},until\underset{\chi \in X_{f}}{⊗}\chi$*. Every advice has a set of constraints* $R_{s}=<{\hat{\tau }}_{d},{\hat{\tau }}_{v},r_{dep}^{s}>$*,* ${\hat{\tau }}_{d}$ *is the maximal process time of the decision that generated from* Ad*,* ${\hat{\tau }}_{v}$ *is the term of validity of* Ad*,* $r_{dep}^{s}$ *is the minimum reliability requirement of the decision.*

Notice that operation instructions op in the composition message for Ob can be a set of operations op={<,≤,=,≥,>,≠,}∪{not occur,occur}. *For safety, one decision contains one final actuator and can only take one action*, because mealy FSM (1) is on not closed status under parallel composition [45], hence, the final action should be processed in serial order. However, it doesn’t says that actuators can’t be the target actor of χ. χ is an observation event, the trigger of a decision can depend on the event whether a target actuator has taken/finished an action.

**Definition 4** (Decision)**.** *Let* $DC=(uuid,Ad,ACT,CP,R_{d})$ *be the decision instance of an advice* Ad*, where* ∀Actor∈ACT *and whose* Tid *is defined in* Ad *or is an network actor;* uuid *is the universally unique identifier,* ∀msg∈M *has the same* uuid *with the decision;* $R_{d}=<\tau_{r},\tau_{sv},r_{dep}^{d}>$ *is the run-time decomposed requirements of* DC. $\tau_{r}={\hat{\tau }}_{d}-\sum \tau_{i}$ *is the remaining processing time of the decision, where* $\tau_{i}$ *is the actual processing time of* $Actor_{i}$*;* $\tau_{sv}=\sum \tau_{i}^{w}-\sum \tau_{i}$ *is the saved time;* $r_{dep}^{d}$ *is the current reliability of decision, which will be introduced in Section 5 and Section 6.1.* ∀msg∈CP.M *belongs to a composition pattern, which will be introduced in Section 5.2. And also* ∀msg∈CP.M *has a time bound* $<\tau_{w},\tau_{rs}>\in T$*,* $Actor_{i+1}$ *first waits for time* $\tau_{w}$ *then starts to process the decision,* $\tau_{w}$ *is the reserved time for parallel composition to synchronize the processing;* $\tau_{rs}$ *is the reserved time for decision process,* $\tau_{rs}={\hat{\tau }}_{d}-\sum \tau_{i+1}^{w}$*. In summary,* $Actor_{i+1}$ *should wait* $\tau_{w}$ *and finish the* (i+1)th *step of decision in* $\tau_{r}-\tau_{rs}=\sum \tau_{i+1}^{w}-\sum \tau_{i}$.

*Uuid* is the identification to avoid repeatedly processing one decision on the same actors, which is an important constraint to prevent duplication and maintain safety. $R_{d}$ is for transmitting the dynamic requirement to successor actors, $\tau_{w}$ is for synchronization, $\tau_{rs}$ is used to control the deadline of process. The example of the formal process flow is introduced in Appendix A.1.

### 4.3. Centralized and Decentralized Decision Process

According to the way of decision management, there are two kinds of decision process forms. One is *centralized decision process*; another is our proposal, *decentralized decision process*. Without loss of generality, the local DSS generates an advice with two $\chi_{s}$, $if\;e_{1}\&e_{2},\;then\;excute\;Actor_{act},\;until\;e_{3}$.

**Figure 9:**
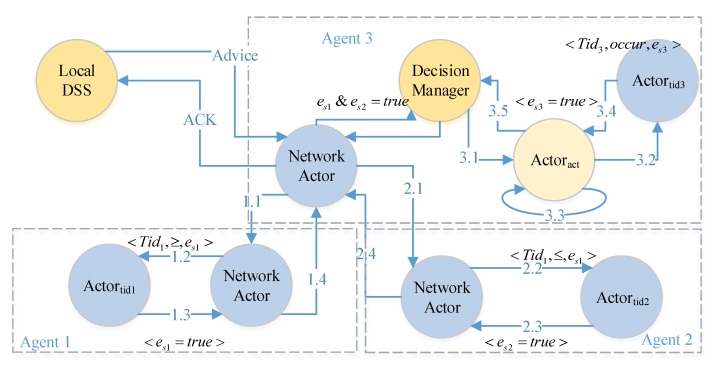
A possible solution for traditional centralized decision process.

The *centralized decision process* flow is illustrated in Figure 9. The local DSS sends an advice to a decision manager and the decision manager controls the flows of a decision process. At every step (1.1 to 1.4, 2.1 to 2.4 and 3.1 to 3.5), the sensors and actuators should acknowledge to the manager, then the manager sends the command for next operation. By the way, $Actor_{act}$ is also a decision manager to the process of $e_{3}$.

**Figure 10:**
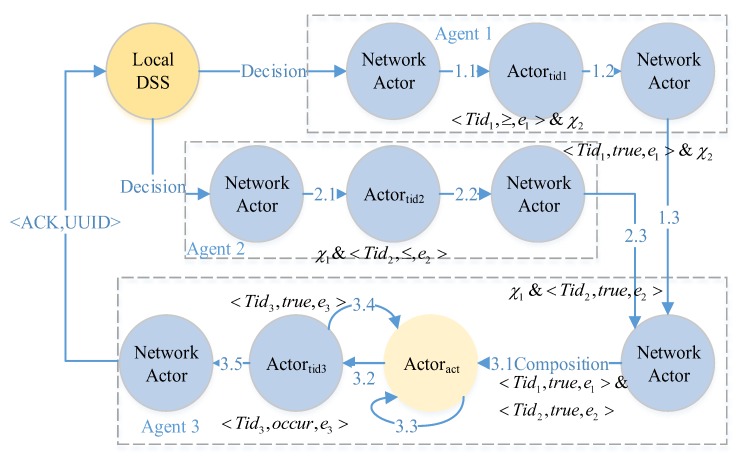
A possible solution for decentralized decision process based on our framework.

To overcome the single point of failure and to minimize the duration (time) error for event observations, we design a decision as a program solution. The *decentralized decision process* flow is illustrated in Figure 10. A decision is processed with the flow of transmission. It has no explicit decision manager. In some sense, every actor can be regarded as a decision manager for next step composition. The successor waits for all messages from its preorders according to the composition pattern (step 3.1). Based on the decentralized solution, CPS can observe the firsthand events (both physical events and cyber events).

### 4.4. Simplify Self-Management Strategies with Self-Similar Actor

CPSs have massive subsystems, and some of them are heterogeneous. It is impossible to specify strategies for every subsystem. In general, most of the subsystems have limited resources, it is too complex to apply enough powerful strategies to adapt to all situations. Moreover, it is also impossible to exhaust all situations. The systematic solution is need to decease the complexity of runtime decision management.

**Figure 11:**
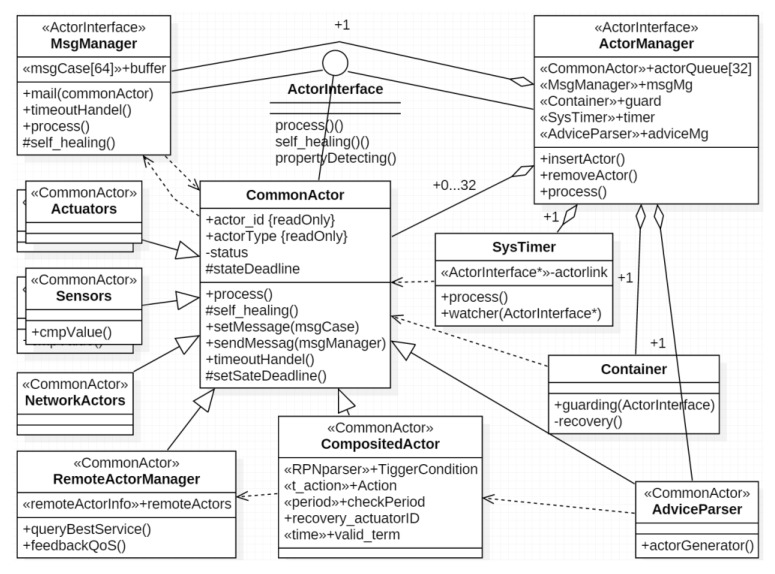
Self-similar actor interface.

The key idea to achieve self-management without deceasing the dependability is using simplicity to control complexity [5] and simplifying the management (control) based on self-similarity [46]. To achieve this, we need to take full advantage of the characteristic of SoS and design a systematic framework and self-similar subsystems to enable recursive composition for CPS. Our framework includes four levels of abstraction: *CPS*, *Agent*, *CompositedActor* and *CommonActor*. The BNF (Backus Normal Form) of the composition relation is shown in Equation (1). To achieve self-similarity, we propose a well-design actor interface to simplify the self-management. These actors share a set of similar operations, the self-similar interface is shown in Figure 11. By applying FSM based actor design, we simplify the constraints for runtime decision decomposition and actor composition. The detailed composition pattern will be discussed in Section 5.

(1) $$\begin{array}{l} CPS::=Agent|CPS\\ Agent::=CompositedActor|CommonActor\\ CompositedActor::=CompositedActor|CommonActor \end{array}$$

Base on the thought of everything as an actor, we can abstract the decision with *compositedactor*, which can be recursive decomposed at runtime. Based on the self-similar interface design, the *ActorManager* on different agents can manage every sub-part of decision with the same rule. And every actor supports a set of same actions *self-healing*() and *property_detecting*(). *property_detecting*() is dedicated to check the requirements with the actors’ properties, which include process time and reliability. A *compositedactor* is generated by the *adviceparser* according to the advice. The *compositedactor* just fills the *Tiggerconditions* if there is not $Actor_{act}$ on the same agent. Otherwise, the *compositedactor* take actions if the value of the Boolean expression of the *Tiggerconditions* is true.

**Figure 12:**
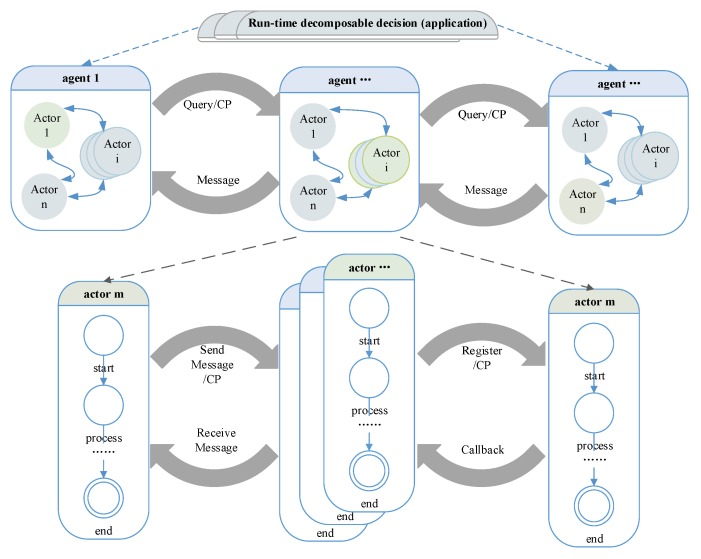
Self-similar dynamic behavior of CPS.

**Figure 13:**
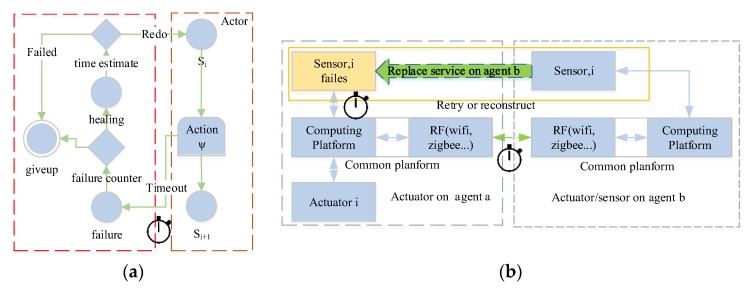
Self-similar healing at actor level and agent level. (**a**) Time based actor level self-healing; (**b**) Time based agent level self-healing.

By using message-based composition, actors share the same communication pattern. Combining with the self-similar interface, actors can have a self-similar behavior, which is depicted in Figure 12. For example, based on the observation event X=<Ob,Tid,X> and CP, the observation is recursive (Boolean operation is closed); logically, any level subsystem can be an observer. The recursive decomposition of event stops when the event is an atomic event, where χ∈Σ. Based on the recursive design, a complex strategy/decision can be decomposed and processed by basic actors. Based on self-similar behavior, simple (self-healing) rules can be applied at all levels of CPS, which is shown in Figure 13. The threshold for the timeout detection are the time bound T which defined in Section 4.2.

## 5. Composition Rules for Reference Framework

As CPS may dynamically reconstruct at any time, different subsystems may be selected to process the decision. Hence, the ACT of one same decision may be entirely different in different executions. Even the physical communication topology and the hardware structure may be dynamic, i.e., the communication topology of the smart fertilization CPS that consists of the unmanned aerial vehicle (UAV) and WSN, or the hardware structure of a Network-on-Chip (NoC) system. The behavior of a decision changes with the actors involved. In this section, we formally analyze the consistency between decision (advice) requirements and subsystem properties based on the reference framework and give the rules for run-time composition to guarantee the correctness and dependability.

### 5.1. Composability and Compositionality of Actors

Improve the C&C of actors is an effective solution without introducing too much complexity. The theory on composability and compositionality for actors are detailed in [47,48]. One main issue that limits the C&C of actor is that the composed actor may have potential deadlocks due to the data flow loop. As every decision and each transition of actor has a deadline (time property), this issue is not so serious. Also, as we analyze in this paper, we just focus on the rules for the composition of properties and runtime requirements decomposition.

#### 5.1.1. The Pattern of Composition

**Figure 14:**
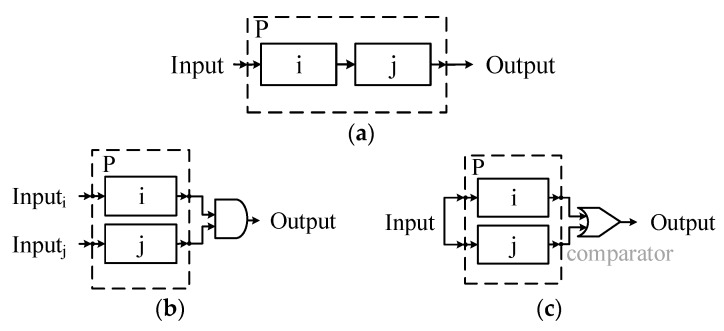
The basic pattern of composition. (**a**) Serial composition; (**b**) Functional parallel composition; (**c**) Redundant composition.

The three basic formats of composition are illustrated in Figure 14. In each format, P is composited with i and j. In Figure 14b, i and j have different functional logic and perform parallel. For redundant composition in Figure 14c, i and j also perform in parallel, but have the same functional logic.

According to the automata theory, FSM is closed under the operations: union, intersection, concatenation, substitution, homomorphism, etc. The composite FSM can generate the identical *trajectory* with the sub-FSMs under the same input (advice), so FSM-based actors are compositional for structures in Figure 14 (*compositedactor* is still an *actor*, it inherit the logical function and interface from the sub-actors. The design of *compositedactor* is shown in Figure 11).

**Figure 15:**
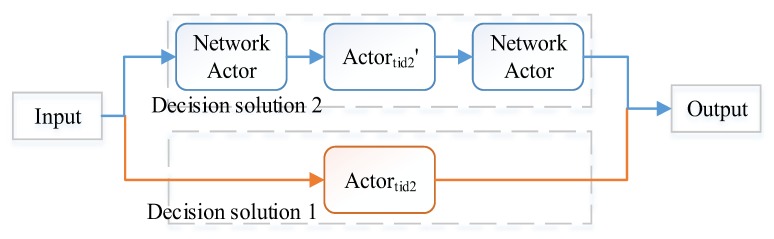
Actor is closed under substitution and concatenation.

Therefore, we can transform dynamically the advice to decision and reconstruct the decision according to the closure of *union, intersection* (composition for parallel observation, Figure 14b), *concatenation* (i.e., hierarchical structure composition, Figure 14a), simplify the QoS-based self-optimization and replacing based self-healing based on *homomorphism* (for replacing with the actors on heterogeneous agents), and *substitution* (replacing the $Actor_{tid2}$ with two $Actor_{net}$ and $Actor_{tid2}\prime$ or building a redundant composition with $Actor_{tid2}$ and $Actor_{tid2}\prime$, Figure 15). We can use the closure of *reversal* to simplify reasoning.

Notice that, Mealy FSM is not closed under parallel composition [45], because the component i and j may depend on each other (cyclic dependency, also called an algebraic loop). To break the cyclic dependency, we limit the amount of final actuator to one (see Section 4.2). FSM is closed just means that the functional logic of FSM (the trajectory of input and output) is closed under these operations. It doesn’t mean that the properties of subsystem are also closed, i.e., the worst case response time is not closed under homomorphism and substitution.

#### 5.1.2. Constraints and Solution for Composability

(1) **Interface composition:** (Compatibility)

According to the interface theory, two interfaces are not compositional, because one can’t accept the error output that generated by another interface [32]. Compositional interface can be achieved by designing uninterruptible self-healing operation and noticing other actors before starting to self-heal, because notification and timeout event are acceptable to all actors. Other actors can reconstruct the decision, so no error output will be generated and sent to other actors. After recovery, the state will be restarted from the state $s_{0}$.

(2) **Consistent transition:** (limits the effects of failures)

Actor is *consistent* in transition, iff Actor produces identical output sequence $\psi_{1}\cdots \psi_{n}$ for all valid input sequences $\varepsilon_{i}\cdots \varepsilon_{m}$, and all $t_{\psi }\leq {\hat{t}}_{\psi }$.

For normal transitions without error states, according to the automata theory, this constraint can be easily complied. If Actor fails, the conclusions can’t be made, because it may generate an erroneous output which is unacceptable to other actors. To an actor, it can’ keep the consistent timing behavior in such situation. The only solution is to apply the redundancy methods which will be introduced in Section 5.2 and Section 6 to minimize the risk of failure. Meanwhile, we use the methods introduced in (1) interface composition to stop all actions immediately until the actors is recovered.

(3) **Composition of actor should be non-commutative:** (for causality, observability and traceability)

$Actor_{i}⊙Actor_{i}\neq Actor_{j}⊙Actor_{i}$

The cyclic dependency of actors will confuse the observation and make decision tracing difficult. The behavior is not inferable, if the composition is commutative because the *trajectory* of $Actor_{i}⊙Actor_{j}$ is the same with $Actor_{j}⊙Actor_{i}$. The observer can’t infer the behavior based on the *trajectory*. Hence, if $Actor_{i}⊙Actor_{i}=Actor_{j}⊙Actor_{i}$, such composition should be forbidden, or $Actor_{j}$ and $Actor_{i}$ should be designed as a one huge actor.

### 5.2. Composition Rules of Reliability and Time (Duration) Properties

The compositionality of dynamic requirements and the composability of properties in dynamic behavior are two sides of the same coin. For DSS, checking the rationality of an advice is estimating the holistic properties of a decision with the properties of the actors. It should take into account the available structures for processing. For the actors who process the decision, evaluating the practicability of dynamic arrangement (decision decomposition) is checking the fitness between run-time requirements with the properties of (next step) actors.

Most requirements/properties of decisions, which include both the system level requirements and subsystem level requirements, change over time. And most properties of subsystems just depend the duration of processing, which can be specified by a function of duration/time, i.e., the reliability R(τ), and energy consumption E(τ)=P×τ. In this paper, we focus on the dependability and process time, but this method can provide the reference for other requirements.

#### 5.2.1. Calculation Rules for Reliability Composition for Relative Time Based Framework

The reliability function is written as $R(\tau )=1-F(\tau )=1-\int_{0}^{\tau }p(\tau )d\tau$, where F(τ) is the failure rate function, p(τ) is the failure density function, τ is the duration, R(τ)∈(0,1). To simplify the equation, we use the absolute duration in this Section (because the process of statistic of p(τ) is based on absolute duration; we can transform it into a relative time model with $R_{0}(\tau_{0})=1-\int_{0}^{c(\tau_{0})}p(c(\tau_{0}))d(c(\tau_{0}))$, where $\tau_{t}=c(\tau_{0})=\tau_{0}\times f_{b}/f_{0}$, $\tau_{t}$ is the duration of target actor, $\tau_{0}$ is the duration of the observer actor. $f_{b}$ is the absolute frequency and $f_{0}$ is the frequency of MCU where observer runs on).

We conclude seven types of composition solutions, which are shown in Table 3. $t_{i}$ refers to the timestamp of last recovery of $Actor_{i}$, $\tau_{i}^{r}$ refers to the elapsed time from last recovery, $\tau_{i}^{p}$ is the process time $\tau_{i}^{b}\leq \tau_{i}^{p}\leq \tau_{i}^{w}$. $t_{i}+\tau_{i}^{r}$ is the timestamp when $Actor_{i}$ starts to process current event and $t_{i}+\tau_{i}^{r}+\tau_{i}^{p}$ is the timestamp when finishes to process current events. To simplify, let $\tau_{i}=\tau_{i}^{r}+\tau_{i}^{p}$, $R_{i}(\tau_{i})=1-F_{i}{|}_{t_{i}+\tau_{i}^{r}}^{t_{i}+\tau_{i}^{r}+\tau_{i}^{p}}$. Notice that, the equations in Table 3 can also be applied as the rules for the decomposition of reliability requirement at run-time.

The pattern 1 and pattern 2 are the two basic functional composition patterns, and the pattern 3 to 5 are the basic redundant processing patterns. All three patterns start m actors simultaneously to process the same decision. Pattern 3 accepts the first returned output without waiting others (i.e., Reliable message transmission). Pattern 4 doesn’t start next action until all actors finish the actions (i.e., guarantee the reliability of sensing data). Pattern 5 starts next action after receiving *k* (same) outputs. Pattern 6 and 7 are composite patterns to tradeoff between time requirement and reliability. CPS can apply different strategies (pattern 3–5) to accept the outputs in every step.

Both reliability and process time are very important to safety-critical CPS. We can apply different composition patterns to arrange the decision process to achieve balance between the dependability, efficient (minimizing the amount of redundant actors) and correctness (to meet the requirement of $\tau_{r}$, in other word, finishing the decision in time). For example the reserved time $Min(\tau_{DC})<\tau_{r}-\tau_{rs}<Max(\tau_{DC})$ and the runtime reliability requirement for the next step is $Min(R_{DC})<r_{dep}^{d}<Max(R_{DC})$, we can apply the pattern 6.2 and with pattern 5 to meet the constraints of time and reliability at the same time.

#### 5.2.2. Constraints and Solutions for Redundant Composition

**(1) Consistent message sending for redundant composition**

For a one-to-many redundant composition $Actor_{p}(s_{i},\psi_{i})\overset{msg}{\to }\{Actor_{i}(s,\psi ),Actor_{j}(s,\psi )\}$, we have $\left(msg(p,i)≜msg(p,j))\wedge (Actor_{i}=Actor_{j}\right)$. Notice that, both space based redundancy (pattern 3, 4, 5) and time based redundancy (redo) should satisfy these constraints.

**(2) Consistent receiving behavior for redundant composition**

For a many-to-one redundant composition $\left\{Actor_{i}(s,\psi ),Actor_{j}(s,\psi )\}\overset{msg}{\to }Actor_{p}(s_{i},\psi_{i}\right)$, we have $\left(msg(i,r)\equiv msg(j,r))\wedge (Actor_{i}=Actor_{j}\right)$.

Where ∧ is the AND operator of Boolean logic.

**(3) Constraints for advices and decisions**

For an operable advice, the requirements of time should meet $\sum_{Actor\in Ad}{\hat{\tau }}_{i}^{b}\leq {\hat{\tau }}_{d}$, (${\hat{\tau }}_{i}^{b}$ is BCET defined in Section 4.2), and the dependability requirements should meet $r_{dep}<Max(R_{DC})$, if $\tau_{rs}<Max(\tau_{DC})$. If $\tau_{rs}>Max(\tau_{DC})$ and $\tau_{d}>{\hat{\tau }}_{i+1}^{w}$, we can try redo $Actor_{i+1}$ to improve the reliability.

## 6. Improving the Reliability of Decision Processes with Dynamic Structure and Dynamic Behavior

### 6.1. Decision Process Patterns and Reliability

We summarized four kinds of decision arrangement solution, two of them are traditional solutions, and another two are designed for our framework. In this subsection, all involved actors are composited actors, and the final composition structure of decision is the same with the structure 1 of Table 3. The availability of n compositional actors is a classical Markov repairable system [49], which is briefly introduced in Appendix A.1.

#### 6.1.1. Static Decision Process Strategy (*Static* for Short)

**Figure 16:**
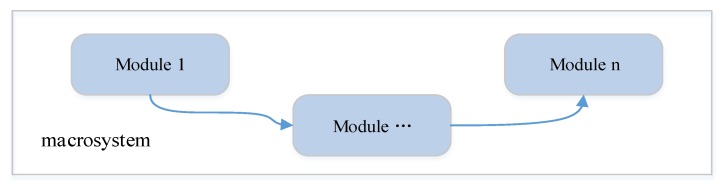
Static processing on macrosystem.

It is the traditional solution for the static architecture, where both hardware and software are centralized. Applications are built as a macro-system. All actors are tightly implemented as a union, and the connections between actors can’t be modified dynamically. The structure is shown in Figure 16.

As all subsystems (components) are built on agent, every subsystem is online. The online duration of subsystem i is $\sum_{j=1}^{i}\tau_{j}$, where $\tau_{j}$ is the decision process duration on $Actor_{j}$. The reliability of a decision using this solution is shown in Equation (2):

(2) $$R^{st}=\prod_{i=1}^{n}R_{i}(\tau_{i})=\prod_{i=1}^{n}(1-\int_{t_{s}}^{t_{s}+\sum_{j=1}^{i}\tau_{j}}p_{i}(\tau )d\tau )$$

#### 6.1.2. Centralized Decision Process Strategy (*Centralized* for Short)

This is a typical decision process flow of the current solution, the hardware structure is decentralized but the control is centralized. The centralized decision manager selects next actors from distributed agents, and controls the flow of the decision process. The structure is shown in Figure 9. For this structure, the centralized decision manager should be online during the whole process time. The reliability function of the decision applying this solution is shown in Equation (3). Notice that, to focus on decision process, we ignore the processing time of decision manager in each step. As a result, $R^{cnt}$ is larger than the real value. In simulation, we let the failure density function of manager $p_{mg}=p_{1}$:

(3) $$R^{cnt}=R_{mg}\times \prod_{i=1}^{n}R_{i}(\tau_{i})=(1-\int_{t_{s}}^{t_{s}+\sum_{i=1}^{n}\tau_{i}}p_{mg}d\tau )\times \prod_{i=1}^{n}R_{i}(\tau_{i})$$

#### 6.1.3. Simple Decentralized Dynamic Decision Process Strategy (*simple-dy* for Short)

The decision is processed dynamically without feedback control, where both structure and control are decentralized. The decision process flow is shown in Figure 10. As actors can heal themselves dynamically, these actors have different online durations ($t_{0}$ of every actor is different). The decision process fails if and only if the actor fails when it is processing the decision. The reliability of a decision with this solution is written as an Equation (4):

(4) $$R^{pre}=\prod_{i=1}^{n}R_{i}(\tau_{i})$$

#### 6.1.4. One-Order Feedback Decentralized Dynamic Decision Process Strategy (*OneOder_dy* for Short)

**Figure 17:**
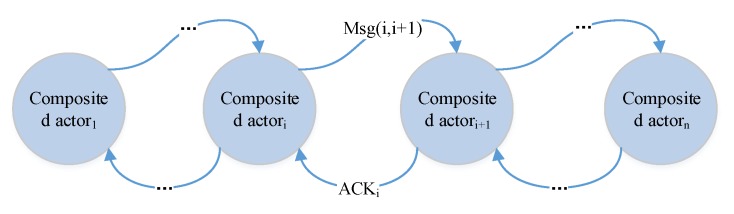
One-order feedback dynamic processing.

The flow of one-order feedback dynamic decision process is illustrated in Figure 17. Suppose that $Actor_{i}$ and $Actor_{i+1}$ are two connected composited actors, and $Actor_{i+1}$ is processing the decision.

(1) If $Actor_{i+1}$ fails when it is processing a decision and $Actor_{i}$ doesn’t receive the ACK message from $Actor_{i+1}$ after the time $\tau_{i+1}^{w}+\tau_{msg(i+1,i)}^{}$, $Actor_{i}$ can resend the msg(i,i+1) to another $Actor_{i+1}\prime$ to re-process the decision if the time requirement permits. The decision can go on being processed correctly.
(2) If both $Actor_{i}$ and $Actor_{i+1}$ succeed, but $Actor_{i}$ doesn’t receive the ACK message because the network failed. The $Actor_{i+1}$ can find a successor $Actor_{i+2}$ to process the decision, $Actor_{i}$ may resend a query to $Actor_{i+1}\prime$. As the decision has uuid, the final actuator will just process the decision once and ignore the decision sent $Actor_{i+1}\prime$. This wastes resources but decision can be processed correctly.
(3) Similar to (2), if $Actor_{i}$ fails and $Actor_{i+1}$ succeeds, $Actor_{i+1}$ can just ignore $Actor_{i}$ and pass the decision to the next successor. In addition, the decision can be processed correctly.
(4) If $Actor_{i+1}$ fails and $Actor_{i}$ also fails, no one has the status of the decision. Obviously, no actors can rearrange the process of this decision. Consequently, the decision fails.

Therefore, for one-order feedback solution, decision fails only when the $Actor_{i}$ and $Actor_{i+1}$ are both failed. It takes $Actor_{i}$ ${\hat{\tau }}_{dl}+\tau_{i+1}^{w}+\tau_{msg(i+1,i)}^{}$ to aware the failure and $\tau_{msg(i,i+1\prime )}^{}$ to resend the message to $Actor_{i+1}\prime$. The decision can go on processing. If the $Actor_{i}$ also fails during ${\hat{\tau }}_{dl}+\tau_{i+1}^{w}+\tau_{msg(i+1,i)}^{}+\tau_{msg(i,i+1\prime )}^{}$, this instance of decision fails. Thus the failure rate is shown in Equation (5), where $t_{i}^{f}=t_{i}+\tau_{i}^{r}+\tau_{i}^{p}$, $\tau_{aw}={\hat{\tau }}_{dl}+\tau_{i+1}^{w}+\tau_{msg(i+1,i)}^{}$, $t_{i+1}^{s}=t_{i+1}+\tau_{i+1}^{r}$. The reliability of dynamic processing strategy is shown in Equation (5):

(5) $$\begin{array}{l} F_{1}^{Dy}=\int_{t_{1}^{s}}^{t_{1}^{s}+\tau_{i}}p_{1}d\tau ;\;|ACT|=1\\ F_{i+1}^{Dy}=F_{i}(\tau_{aw})\times F_{i+1}(\tau_{i+1}^{})=(\int_{t_{i}^{f}}^{t_{i}^{f}+\tau_{aw}}p_{i}d\tau )\times (\int_{t_{i+1}^{s}}^{t_{i+1}^{s}+\tau_{i+1}^{}}p_{i+1}d\tau );\;|ACT|>1 \end{array}$$

(6) $$R^{Dy}=\{\begin{array}{c} 1-\int_{t_{1}^{s}}^{t_{1}^{s}+\tau_{1}^{}}p_{1}d\tau ;\;|ACT|=1\\ (1-\int_{t_{1}^{s}}^{t_{1}^{s}+\tau_{1}^{}}p_{1}d\tau )\times \prod_{i=2}^{n}\left(1-F_{i}^{Dy}\right);\;|ACT|>1 \end{array}$$

Notice that, if $\tau_{i+1}^{w}+\tau_{msg(i+1,i)}^{}<{\hat{\tau }}_{dl}$ can’t be satisfied, $Actor_{i}$ has no time to find another available actor, the decision fails; but we can decrease the failure possibility by redundant solution, i.e., arranging more successors to process the decision, which was introduced in Section 5.2.1. By the way, we can also develop a high-order system to improve the reliability, the ACK message should be sent from $Actor_{i+1}$ to $Actor_{i}$ and to $Actor_{i-k}$ in recursive form, so $Actor_{i-k}$ also knows the statues of the decision.

### 6.2. Simulation and Result Analysis

According to the Equations (2)–(4) and (6), we reach the conclusion that the reliability decreases with the process time, failure aware time (WCET), because reliability value F(t)<0. Here, we conduct a set of simulations with MATLAB to test reliability of the four strategies of decision process against the complexity of decision (the amount of composited actors).

In this simulation, we assume that the failure functions of all actors are following the *exponential* distribution ($F(t)=1-e^{-\lambda t}$), which is a common assumption for reliability evaluation. The failure rate λ of each actors fallows uniform distribution, whose range is [0, 0.0002]. The amount of actors increases from 2 to 40 with step 2. The process time τ of each actor follows uniform distribution, whose range is [100, 300]. The range of process time affects the Min reliability and Max reliability of process, and the more results of different ranges is shown in Appendix A.3. The WCET $\tau_{}^{w}=1.1\times \tau$; the ${\hat{\tau }}_{dl}=20$; $t_{i}^{s}=0$ which means that all actors are renewed for decision process. The URL for scripts and simulation data is in supplementary materials.

**Figure 18:**
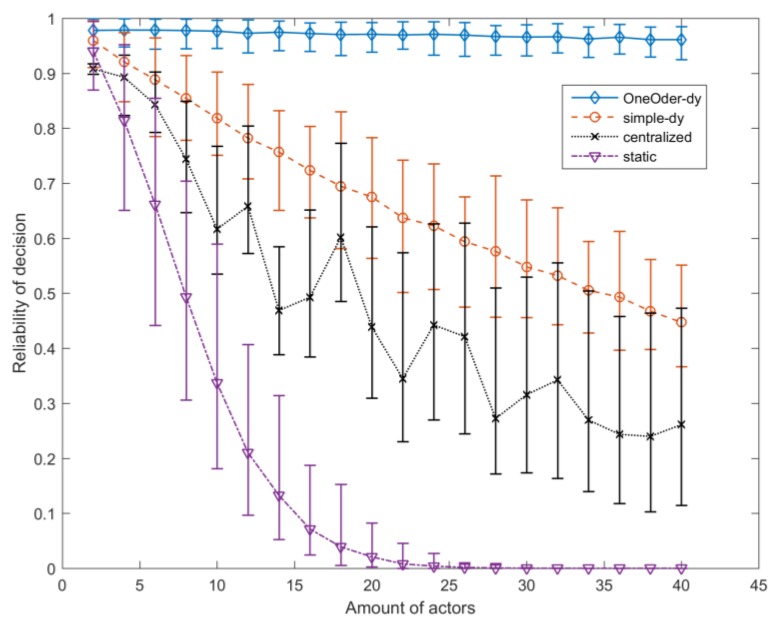
The Mean, Max and Min reliability of four strategies against the complexity of decision, (for centralized decision process, we set $p_{mg}=p_{1}$).

**Figure 19:**
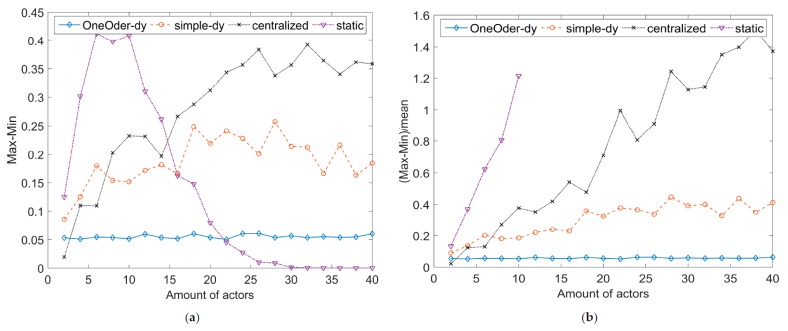
Stability of reliability under random failure and process time (As the curve of *static process* increase much faster than other strategies, it just has the first 5 points in Figure 19b). (**a**) Max−Min of the reliability; (**b**) (Max−Min)/Mean.

For each amount of actors, we simulate 100 times, where λ and τ are the same for the four strategies. In each simulation under same amount of actors, λ and τ are renewed. The simulation results of four strategies against the complexity of decision (the amount of actors) are illustrated in Figure 18. Furthermore, the part of statistic results are listed in Table 4. The stability analysis is shown in Figure 19.

The obtained simulation results show that dynamic, decentralized decision process can achieve not only higher reliability (*Static process <* *Centralized process < Simple dynamic process < one-order feedback*) but also higher stability and higher scalability. *Static process* is centralized control with centralized structure; the *centralized process* is centralized control with decentralized structure; *simple dynamic process* and *one-order feedback* are decentralized control with decentralized structure. The curves in Figure 18 show that decentralization can increase the reliability of the system. *One-order feedback* solution has the highest reliability. For each amount of actors, *one-order feedback* solution also has smaller value of Max−Min and (Max−Min)/Mean, which shows that it has more stable behavior against the variable process time. Thus, the reliability of *one-order feedback* is more predictable, and it is important for decision arrangement. With the increasing of complexity, the values of Max−Min and (Max−Min)/Mean show that one-order feedback solution is more stable. The reliability of one-order feedback solution decreases slowly with the amount of actors, which shows higher scalability. It means that one-order feedback solution can be applied for more complex decision and involves more actors. In addition, *one-order feedback* *strategy* can achieve higher reliability and stability than *simple dynamic decision process strategy* without introducing time overhead (it just increases the memory overhead, because the preorder actor should keep the message until the successor actor returns the ACK).

### 6.3. Proactive Self-Healing for Fault Prevention (Risk Management)

There are two types of self-healing strategy: (1) self-healing actions only occur after an actor has failed. Such system is a classical Markov repairable system (as seen in Appendix A.1); (2) The actors take proactive actions to heal themselves before any fault occurs (i.e., an actor can periodically check and restart itself to prevent the faults). CPS is safety-critical. Applying the first self-healing strategy increases risk of missing deadline, because repairing takes time. Therefore, we can apply the second strategy to prevent failures to improve the safety.

According to the hazard function $h_{i+1}(t)=\frac{p(t)}{R(t)}=\frac{R_{i}(\tau_{i})-R_{i+1}(\tau_{i+1}+\Delta \tau_{i+1})}{\Delta \tau_{i+1}\times R_{i}(\tau_{i})}$, we can replace the $R_{i}(\tau_{i})$ and $R_{i+1}(\tau_{i+1})$ with the Equations (2)–(4) and (6), and can get the hazard function of failure for each strategy when $Actor_{i}$ hinds over the decision process to $Actor_{j}$ (the detail equation is attached in Appendix A.2). Obviously, the hazard increasing with the online time of $Actor_{j}$ (*Centralized processing* also depends on online time of decision manager, One-order feedback strategy also depends on the online time of $Actor_{i}$).

We can define a threshold of risk $H_{threshold}$, let $h_{i+1}(\tau )\leq H_{threshold}$, by solving the equation, we can have a $\tau^{p}$ which is the period of self-healing. Therefore, we can control the failure risk and to improve the safety of the decision process. In addition, let us assume that it takes an actor $\tau^{h}$ time to self-heal itself. Thus, the availability of every actor is $A=\tau^{p}/(\tau^{p}+\tau^{h})$.

## 7. Case Study

**Figure 20:**
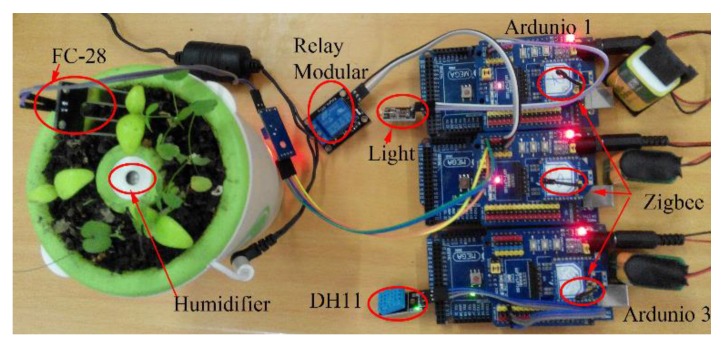
Platform of decentralized decision process of case 2 (to simplify, PC is not included).

To test, validate and evaluate the propose concepts, we implemented a test-bed platform. We used a PC as a local DSS, which connects with other subsystems with a USB to ZigBee adapter. There are three types of Arduino boards (Mega2560) and a humidifier. Type1 (top): the board has a light sensor (Keyes K853518). Type2 (middle): the board has a soil moisture sensor (FC−28) and controls the humidifier with a relay module. And Type 3 (bottom): the board has a temperature and a humidity sensor (DH11). The three types of Arduino cooperate together to process the decision. The platform of case 1 is shown in Figure 20.

In all cases, the actors of the same sensors and actuators are implemented with the same code. We automatically inject the faults on every Arduino board when actors are active, and the frequency of fault injection is one error every 4 s. The subsystem can self-recover from the failures with a container-based multilevel self-healing solution, which is introduced in our previous paper [50].

### Case 1: All actors in one board

It is a macrosystem solution, all sensors and actuators are integrated into one board. It is also a centralized control solution, all process flows are controlled by one agent. Notice that, to improve the reliability, the actuator will notify local DSS the progress of humidifying every one minute

### Case 2: T1_one + T2_one + T3_one

In this case, CPS has no redundant subsystems. It just has one type1 board, one type2 board and one type3 board.

### Case 3: T1_two + T2_one + T3_two

In this case, CPS has two redundant type1 boards and two redundant type3 boards. It has one type2 board. To avoid over modifying the environment, the process of the final step of decision (humidifying) is mutually exclusive, and only one actuator is in charge of the final step of action.

Two types of failures are injected: (1) actor level failure, which is WCET violation [50]; the target actor will start the self-healing action. Other actors on the same board work normally; (2) Board level failure, which is Random PC Error [50]; the board will be restarted and all actors on this board are failed. The tests take 12 days (it takes about two days to test every case and each failure, from 15 August to 27 August). The deadline of decision process is 15 min. It takes the board 1–4 s to recover from board level failure, and it takes about 80–110 milliseconds to recover from an actor level failure. The results are shown in Table 5 and Table 6.

The failure rates in Table 4 show that the decentralized framework (cases 2 and 3) can tolerate the higher frequency failures, especially board level failure. One-order feedback strategy on decentralized framework (cases 2 and 3 in Table 4) can successfully process all decisions in time. The mean process time (MPT_N) in Table 5 shows that case1 has highest, performance in normal model, but it is not much better than the distributed framework (the overhead of the distributed framework in normal mode is 239.2 − 235.8 = 3.4 s). The mean process time under fault injection (MPT_F) shows that the distributed framework has significantly higher performance than centralized solution. One-order feedback strategy on the redundant decentralized framework (case 3 in Table 5) can shorten the redoing time, cover the time cost of self-healing and improve the dependability of decision process. In summary, One-order feedback strategy on the redundant decentralized framework can tolerate failures, leave more time to actuator to take the final action, which can improve the safety of decision process (the actuator can cautiously process the final action with more frequent checking).

Notice that, to improve the dependability of real world CPS, we have applied multi-level measures, which include actor level self-healing solution, node level self-healing solution, decentralized fault detection solution, etc. The results in Table 4 are the comprehensive effect of these measures. Moreover, the duration of process time is affected by the weather (the humidity and temperature). Normally, it takes the humidifier about 4 min to increase the moisture from 30 to 50 (the moisture of surface soil has been increased to 50). In addition, it takes about 15 min to dry the soil moisture from 50 to 30 in Harbin in August.

## 8. Discussion

In this paper, we mainly focus on the introduction of a compositional framework and the evaluation of decentralized decision process. A CPS is an autonomic computing system which should be able to adapt to the changeable environment, prevent and recover from various failures automatically. To achieve this goal, CPS has to adjust its structure and behavior dynamically. In this paper, we introduce a systemic solution to improve the consistency of event observation (the long-term loop) and the dependability of decision process (the short-term loop). To solve the inconsistent timestamp of the events, an observer based relative time solution is proposed to guarantee the consistent event observation for causal reasoning and processing duration management. The relative time solution infers the timestamp when the events occur with process duration and the timestamp that event observed. Using the locality of events, we can select the nearest local observer to control the errors of observation. This solution doesn’t need the global reference time and periodic clock synchronization, it can increase the scalability of CPS.

To minimize the errors of observation and to overcome single point failure of centralized decision process, we design a formal reference framework based on compositional actor for self-management CPS. Base on the thought of decision as a program, actor-based decisions (advice) can be decomposed and composed at runtime. Moreover, a self-similar recursive actor interface is proposed to simplify self-management. We provide the patterns and evaluation rules and constraints for reliability and process time composition and decomposition.

Based on this framework, we propose a *simple dynamic decision process strategy* and a *one-order dynamic feedback decision process strategy* and compare the reliability with traditional static strategy and centralized decision process strategy, the simulation results shows that the one-order dynamic feedback strategy has high reliability, scalability and stability against the complexity of decision and random failure.

The testing results of the real world system show the comprehensive improvement of dependability with our framework. Our compositional framework improves the scalability through three main solutions: (1) the relative time model is applied to remove the central reference time node; (2) the compositional framework supports decentralized decision process; (3) one-order dynamic feedback strategy improves the scalability. CPS can apply different composition patterns to achieve the balance between requirements of safety, reliability and process time.

In this paper, we show a way to simplify the dependability evaluation for dynamic systems. By improving the composability and compositionality of actors, we can evaluate the system requirements with the properties of compositional actors, and deduce the system behavior from the behavior of subsystems, which can accelerate the progress of evaluation significantly.

## Figures and Tables

**Table 1:** Comparison between absolute time model and relative time model.

	Absolute Time Model	Relative Time Model
Frequency of synchronization	Periodically sync	Once
Error of timing	Increases with the time during a synchronizing period and increase with hops (synchronization error)	Increase with the hops between event source and observer (no more than the error of absolute time) ^1^
Global reference time	Need	Not necessary
Scalability	It depends on the scalability of the synchronized algorithm	High

^1^ Because the relative time mode uses the same method to estimate the network transmission time. Indeed the relative time model can estimate in each communication time and use the mean value to remove the error.

**Table 2:** Three types of communication for definition.

Communication Patterns	Illustrations
$Actor_{i}(s_{k},\psi_{k})\overset{msg}{\to }Actor_{j}(s_{m},\psi_{m})$ Point-to-Point	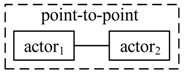
$Actor_{i}(s_{k},\psi_{k})\overset{msg}{\to }\{Actor_{j}(s_{m},\psi_{m})\}$ One-to-Many	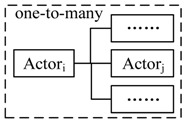
$\left\{Actor_{i}(s_{k},\psi_{k})\}\overset{msg}{\to }Actor_{j}(s_{m},\psi_{m}\right)$ Many-to One	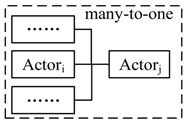

**Table 3:** The composition rules for reliability and duration.

Patterns	The Structure of the Composition	$R_{DC}(\tau )$, $\tau_{DC}$ and t^1^
(1) Basic pattern	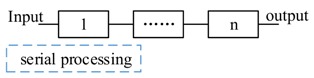	$\prod_{i=1}^{n}R_{i}(\tau_{i}^{o}+\tau_{i})$, where $\tau_{DC}=\sum_{i=1}^{n}\tau_{i}$, $t_{1}+\tau_{1}≺\cdots ≺t_{n}+\tau_{n}=t$
(2) Parallel pattern (time critical)	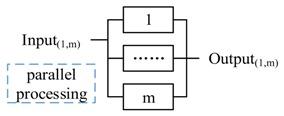	$\prod_{i=1}^{m}R_{i}(\tau_{i})$, where $\tau_{DC}=\tau_{i}$, i is the index of the $\max_{i=1}^{m}\{t_{i}+\tau_{i}\}=t$
(3) First wins (time critical)	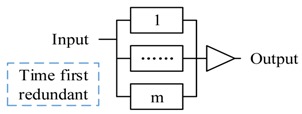	$R_{i}(\tau_{i})$, where $\tau_{DC}=\tau_{i}$, i is the index of the $\min_{i=1}^{m}\{t_{i}+\tau_{i}\}=t$
(4) Check all (reliability critical)	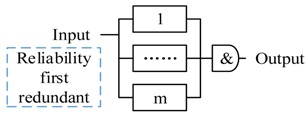	$1-\prod_{i=1}^{m}\left(1-R_{i}(\tau_{i})\right)$, where $\tau_{DC}=\tau_{i}$, i is the index of the $\max_{i=1}^{m}\{t_{i}+\tau_{i}\}=t$
(5) k-Majority (safety critical)	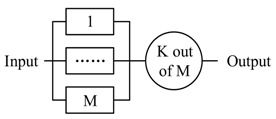	$\sum_{i=k}^{N}\left(C_{n}^{k}(\prod_{i=1}^{k}R_{i}(\tau_{i})\prod_{j=k+1}^{N}\left(1-R_{j}(\tau_{j}))\right)\right),$ where K≤k≤M and $\max_{i=1}^{K}\{t_{i}+\tau_{i}\}=t$, $\tau_{DC}=\tau_{i}$
(6) Hybrid pattern	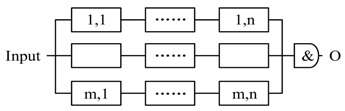 (6.1 Reliability critical)	$1-\prod_{i=1}^{m}\left(1-\prod_{j=1}^{n}R_{i,j}(\tau_{i,j})\right)$, where, $\max\{t_{1,n}+\tau_{1,n},\cdots ,t_{m,n}+\tau_{m,n}\}=t$, $\tau_{DC}=\max_{i=1}^{m}\{\sum_{j=1}^{n}\tau_{i,j}\}$
	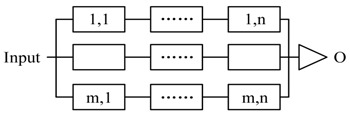 (6.2 Time critical)	$\prod_{j=1}^{n}R_{i,j}(\tau_{i,j})$, where $\sum_{j=1}^{n}t_{i,j}+\tau_{i,j}=t$, $\tau_{DC}=\sum_{j=1}^{n}\tau_{i,j}$
(7) Universal pattern	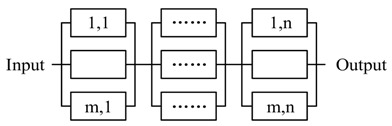 (the structure depends on the type of pattern applied in every step)	$Max(R_{DC})=\prod_{i=1}^{m}\left(1-\prod_{j=1}^{n}\left(1-R_{i,j}(\tau_{i,j}\right))\right)$, $Max(\tau_{DC})=\sum_{i=1}^{n}\max_{i=1}^{m}\{\tau_{i}\}$, (apply pattern 4 in all steps)
		$Min(\tau_{DC})=\sum_{i=1}^{n}\min_{i=1}^{m}\{\tau_{i}\}$, $Min(R_{DC})=\prod_{i=1}^{n}R_{j,i}(\tau_{j,i})$, j is the first actor (apply pattern 3 in all steps)

^1^ $R_{DC}(\tau )$ is the reliability of decision, $\tau_{DC}$ is the duration of decision t is an absolute timestamp when the decision is finished.

**Table 4:** Robustness on reliability of four strategies.

	Mean	Max	MIN	(Max−Min)/Mean ^1^
Static process	0.00000047	0.00000798	0.00000000	16.89455532
Centralized process	0.26113862	0.47278942	0.11442376	1.372319661
Simple Dynamic process	0.44719988	0.55121109	0.36700843	0.411902301
One-order feedback	0.96111892	0.98509618	0.92474253	0.062795188

^1^ The value of Mean, Max and Min are the last case of last simulation (actor = 40) (data file is d0_2-40.mat).

**Table 5:** The failure rates of the three cases.

	Actor Level Failure			Board Level Failure		
	Success	Failed	Failed Rate (%)	Success	Failed	Failed Rate (%)
*Case 1*	190	2	1.04	111	81	42.2
*Case 2*	192	0	0	192	0	0
*Case 3*	192	0	0	192	0	0

**Table 6:** The mean process time and overhead of the three cases.

	MPT_N (s)	Actor Level Failure		Board Level Failure	
		MPT_F (s)	Time Overhead ^a^	MPT_F (s)	Time Overhead
*Case 1*	235.8	512.4	217.3%, 276.6	694.2	294.4%, 458.4
*Case 2*	238.7	407.3	170.6%, 168.6	505.2	211.6%, 266.5
*Case 3*	239.2	254.8	106.5%, 15.6	463.6	181.9%, 224.4

^a^ Format: (MPT_F/MPT_N, MPT_F − MPT_...N).

**Figure A1:**
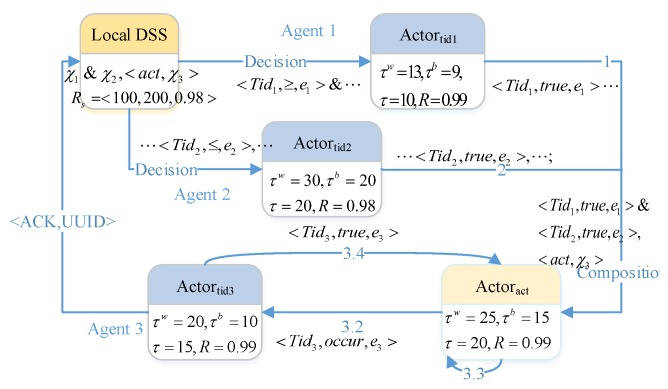
Simplified decentralized decision process.

**Figure A2:**
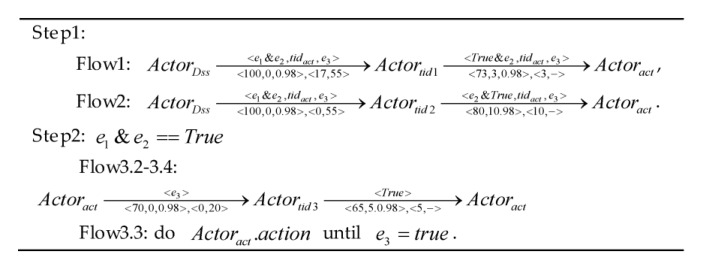
The detail process flow for Figure A1.

**Figure A3:**
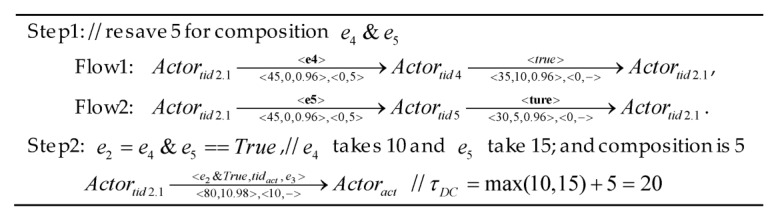
The example flow of decomposition.

**Figure A4:**
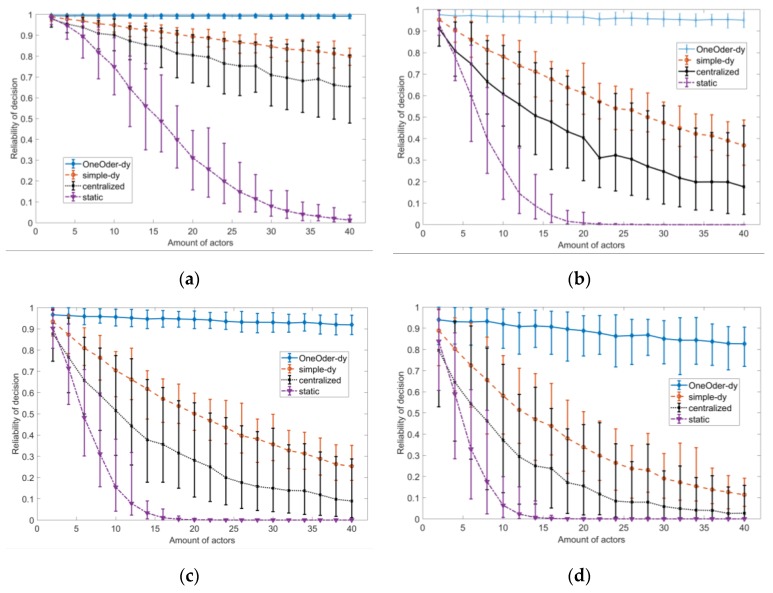
The reliability over different ranges of process time. (**a**) The reliability over the time range [10, 100]; (**b**) The reliability over the time range [200, 300]; (**c**) The reliability over the time range [300, 400]; (**d**) The reliability over the range [100, 1000].

## Supplementary Materials

The simulation code and the record for this figure are available on https://bitbucket.org/Teampp/sensors2017.

## Appendix A

### Appendix A.1. A Case of Decentralized Decision Processing

Here, we use the same advice case that is introduced in Section 4.3. To simplify, we removed the network actor. All properties are shown in Figure A1, and the formal process flow of this example is shown in Figure A2. I.e., the requirement of decision is $R_{s}=<{\hat{\tau }}_{d},{\hat{\tau }}_{v},r_{dep}^{s}>=<100,200,0.98>$. The WCET of $Actor_{tid1}$ is $\tau^{w}=13$, the BCET is $\tau^{b}=9$, the real time of decision processing is τ=10, the reliability of $Actor_{tid1}$ is R=0.99. The format of decision process is $Actor_{i}\overset{<decision>}{\underset{R_{d},T_{msg}}{\to }}Actor_{j}$, where $R_{d}=<\tau_{r},\tau_{sv},r_{dep}^{d}>$, $T_{msg}=<\tau_{w},\tau_{rs}>$ is the time bound of message <decision>, $\tau_{sv}=\sum_{i=0}^{k}\tau_{i}^{w}-\sum_{i=0}^{k}\tau_{i}$, $\tau_{rs}=\tau_{r}-\sum_{i=k+1}^{n}\tau_{i}^{w}$.

Flow1 and Flow2 have a parallel composition. Thus the reliability of $Actor_{tid1}$ and $Actor_{tid1}$ should be larger than 0.98. To synchronize observation, $Actor_{tid1}$ first waits $\tau_{w}=17$ then starts its observation. Let us assume that $Actor_{act}$ receives the message from $Actor_{tid1}$ at local timestamp t. According to the relative time model, $Actor_{act}$ can deduce that event from $Actor_{tid1}$ occurs at t−10, that event from $Actor_{tid2}$ occurs at t−20. $Actor_{act}$ have the right order of event. Such decentralized solution can reduce the error of difference of timestamp.

Without loss of generality, let us assume that $Actor_{tid2}$ is a composited actor, which means that no Actor can meet the reliability requirement. We can improve the reliability with redundant composition which introduced in Section 5.1. If we apply the composition pattern 4, the decomposition rule is $1-\prod_{i=1}^{m}\left(1-R_{i}(\tau_{i})\right)\geq 0.98$, and the maximum $\tau_{DC}<100-55=45$. Thus, local DSS will select m Actor whose tid=tid2 to observe the event $e_{2}$ together. Let us suppose that two *Actors* with tid=tid2 are selected to observe the events together. The reliability of $Actor_{tid2.1}$ is 0.96 and $Actor_{tid2.2}$ is 0.86. 1−(1−0.96)×(1−0.86)=0.9944≥0.98.

As events and actors support recursive composition, every composited actor can decompose the requirement as the local DSS dose in the example. Suppose $e_{2}=<ob,tid_{4},e_{4}>\&<ob,tid_{5},e_{5}>$ is a composited event, $e_{4}$ is atomic event, $e_{4}\in \Sigma$. The decomposed requirement of $Actor_{tid2.1}$ is $R_{s}=<{\hat{\tau }}_{d},{\hat{\tau }}_{v},r_{dep}^{s}>=<45,0,0.96>$. The $Actor_{tid2.1}$ plays as a local DSS, the process flow is shown in Figure A3, replacing the $e_{2}$ with $e_{4}$.

### Appendix A.2. The Availability of Decentralized Framework

The behavior of the n compositional actors is a classical repairable system [49] with k backup components (1≤k≤n), where n−k is the minimum amount of redundant actors to guarantee the reliability ($R_{DC}>R_{requirement}$), the k actors can take over the processing if any actor fails. Without loss of generality, let us suppose that ${\left\{Actor\right\}}_{i}$ is the n compositional actors for the $i^{th}$ step of a decision. $\lambda_{i,j}$ is the transfer rate of $Actor_{i,j}$, $\mu_{i,j}$ is the repair rate of $Actor_{i,j}$.

If all the actors have the same λ and μ (isomorphism), it is a classical Markov repairable system. The transfer matrix $A_{\left(n+1)\times (n+1\right)}$ is a tridiagonal matrix, which is shown in Equation (A1). The stable availability of ${\left\{Actor\right\}}_{i}$ is $A=1-\pi_{n}$, mean up time (MUT) is $\frac{1-\pi_{n}}{\pi_{n}k\mu }$, mean down time(MDT) is $\frac{1}{k\mu }$, mean cycle time is $\frac{1}{\pi_{n}k\mu }$, where $\pi_{n}$ is shown in Equation (A2):

(A1) $$A_{n+1,n+1}=\left[\begin{array}{cccccc} -n\lambda & n\lambda & & & & 0\\ \mu & -(n-1)\lambda -\mu & (n-1)\lambda & & & \\ & 2\mu & -(n-2)\lambda -\mu & (n-2)\lambda & & \\ & \ddots & \ddots & \ddots & & \\ & & k\mu & -(n-k)\lambda -k\mu & (n-k)\lambda & \\ & & \ddots & \ddots & \ddots & \\ & & & k\mu & -\lambda -k\mu & \lambda \\ 0 & & & & k\mu & -k\mu \end{array}\right]$$

(A2) $$\pi_{j}=\{\begin{array}{c} {\left[{\sum_{i=0}^{k}C_{n}^{i}\left(\frac{\lambda }{\mu }\right)}^{i}+\sum_{i=k+1}^{n}\frac{n!}{(n-i)!k!k^{i-k}}{\left(\frac{\lambda }{\mu }\right)}^{i}\right]}^{-1},j=0\\ C_{n}^{j}{\left(\frac{\lambda }{\mu }\right)}^{j}\pi_{0}\begin{array}{ccc} \begin{array}{ccc} \begin{array}{ccc} , & j=1,2,\cdots ,k & \end{array} & & \end{array} & & \end{array}\\ \frac{n!}{(n-j)!k!k^{j-k}}{\left(\frac{\lambda }{\mu }\right)}^{j}\pi_{0}\begin{array}{cc} , & j=k+1,\cdots ,n \end{array} \end{array}$$

### Appendix A.3. The Reliability over Different Rages of Process Time

The reliability decreases with the process time, failure aware time (WCET), (1) The reliability function $R_{i}(\tau_{i})$ is a monotonic decreasing function of the duration $\tau_{i}$; (2) The process of a decision consists several actors. The range of the reliability of each actors is (0, 1). The four Equations (2)–(4) and (6) can be simplified as $R_{dc}=c\times \prod_{i=1}^{n}R_{i}$, where $R_{i}\in (0,1)$. $R_{dc}$ decreases if $R_{i}$ decreases ($R_{1}\times R_{2}<\min(R_{1},R_{2})$, where $0<R_{1}<1,0<R_{2}<1$). As $R_{i}$ decreases with the process time, $R_{dc}$ decreases the process time of decision is $\Sigma \tau_{i}$.

All the parameters used are the same with the simulation in Section 6.2. The actor amount is [2,3,4,5,6,7,8,9,10,11,12,13,14,15,16,17,18,19,20,21,22,23,24,25,26,27,28,29,30,31,32,33,34,35,36,37,38,39,40] with the step of 2. According to the four figures Figure A4a–d. we can make the conclusion that the range of process time affects the Min reliability and Max reliability of process. One-order feedback strategy has best performance of reliability and stability. Obviously, the actor with small process range is more stable (Figure A4a vs. Figure A4d).

### Appendix A.4. Hazard Function of Four Strategies

Hazard function of the static decision process strategy:

$$h_{i+1}^{st}(t)=\frac{R_{i}(t)(1-R_{i+1}(t+\Delta \tau ))}{\Delta \tau_{i+1}\times R_{i}(t)}=\frac{F_{i+1}(\tau_{i+1}^{})}{\Delta \tau_{i+1}}$$

Hazard function of the centralized decision process strategy:

$$h_{i+1}^{cnt}(t)=\frac{R_{mg}^{i}(\sum_{i=1}^{i}\tau_{i})-R_{mg}^{i+1}(\sum_{i=1}^{i+1}\tau_{i})\times R_{i+1}}{\Delta \tau_{i+1}\times R_{mg}^{i}(\sum_{i=1}^{i}\tau_{i})}$$

Hazard function of simple decentralized decision dynamic processing strategy:

$$h_{i+1}^{pre}(t)=\frac{\left(1-R_{i+1}(t_{l.s}+\Delta \tau_{i+1})\right)}{\Delta \tau_{i+1}}=\frac{F_{i+1}(\tau_{i+1}^{})}{\Delta \tau_{i+1}}$$

Hazard function of one-order feedback decentralized decision dynamic processing strategy.

$$h_{i+1}^{dy}(t)=\frac{F_{i}(\tau_{aw})\times F_{i+1}(\tau_{i+1}^{})}{\Delta \tau_{i+1}}$$
